# Heterogeneous Catalytic Upgrading of Biofuranic Aldehydes to Alcohols

**DOI:** 10.3389/fchem.2019.00529

**Published:** 2019-07-26

**Authors:** Jingxuan Long, Yufei Xu, Wenfeng Zhao, Hu Li, Song Yang

**Affiliations:** State Key Laboratory Breeding Base of Green Pesticide and Agricultural Bioengineering, Key Laboratory of Green Pesticide and Agricultural Bioengineering, State-Local Joint Laboratory for Comprehensive Utilization of Biomass, Center for R&D of Fine Chemicals, Ministry of Education, Guizhou University, Guiyang, China

**Keywords:** heterogeneous catalysis, biomass conversion, carbohydrates, furfural, 5-hydroxymethylfurfural

## Abstract

Heterogeneous catalytic conversion of lignocellulosic components into valuable chemicals and biofuels is one of the promising ways for biomass valorization, which well meets green chemistry metrics, and can alleviate environmental and economic issues caused by the rapid depletion of fossil fuels. Among the identified biomass derivatives, furfural (FF) and 5-hydroxymethylfurfural (HMF) stand out as rich building blocks and can be directly produced from pentose and hexose sugars, respectively. In the past decades, much attention has been attracted to the selective hydrogenation of FF and 5-hydroxymethylfurfural using various heterogeneous catalysts. This review evaluates the recent progress of developing different heterogeneous catalytic materials, such as noble/non-noble metal particles, solid acids/bases, and alkali metal salts, for the efficient reduction of bio-based furanic aldehydes to alcohols. Emphasis is laid on the insights and challenges encountered in those biomass transformation processes, along with the focus on the understanding of reaction mechanisms to clarify the catalytic role of specific active species. Brief outlook is also made for further optimization of the catalytic systems and processes for the upgrading of biofuranic compounds.

## Introduction

Selective conversion of reproducible bio-resources into fuels and valuable chemicals has caused great attention because of the rapid economic development of world accompanying with the ever-increasing demand for energy from fossil resources (Li et al., 2018a,b; He et al., 2019a). In this regard, lignocellulose, one of the most abundant and inexpensive carbon sources, is comprised of crucial polymers including cellulose, hemicellulose, and lignin. Among of them, cellulose and hemicellulose can be easily converted into biofuels and platform molecules by hydrolysis and subsequent reactions (Petrus and Noordermeer, 2006; Corma et al., 2007; Serrano-Ruiz et al., 2011; Sankar et al., 2012; Besson et al., 2013; Cai et al., 2014a; Climent et al., 2014; Liu and Zhang, 2015; De et al., 2016a,b; Li et al., 2016b; Zhou and Zhang, 2016).

**Figure 1:**
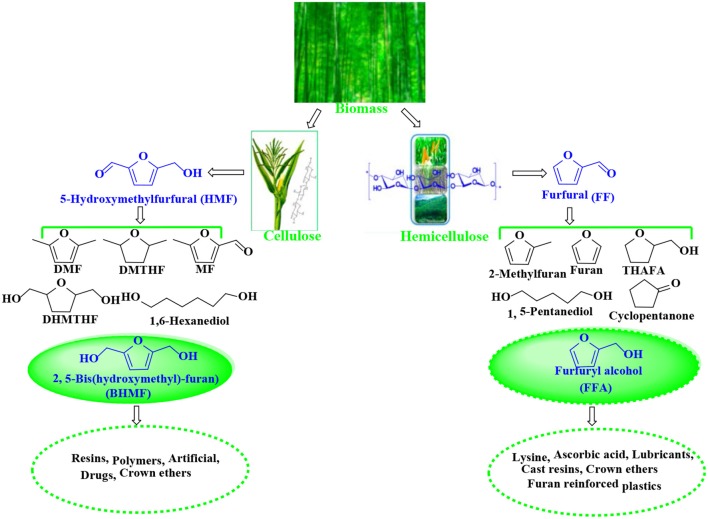
Catalytic upgrading of biomass-based HMF and FF into various value-added products. THAFA, tetrahydrofurfuryl alcohol; FFA, furfuryl alcohol; DMF, 2,5-dimethylfuran; DMTHF, 2,5-dimethoxytetrahydrofuran; MF, 5-methylfurfural; DHMTHF, 2,5-dihydroxymethyl-tetrahydrofuran; BHMF, 2,5-bis(hydroxymethyl)-furan.

5-Hydroxymethylfurfural (HMF) and furfural (FF), known as significant platform molecules, are obtained by hydrolysis/dehydration of cellulose and hemicellulose via the specific catalysis (Wang et al., 2018). FF can be converted into many useful chemicals via hydrogenation (Seo and Chon, 1981; Sitthisa and Resasco, 2011; Chen et al., 2012; Hronec et al., 2012), such as tetrahydrofurfuryl alcohol, 1,5-pentanediol, furan, 2-methylfuran, cyclopentanone, and furfuryl alcohol(FFA), as shown in Figure 1. FFA has received much attention due to its wide range of applications. For example, FFA can serve as low-cost raw material to synthesize lysine, ascorbic acid, plastics, common resins, and lubricants (Chen et al., 2002; Khan et al., 2011; Yan et al., 2014; Yuan et al., 2015a; Shirvani et al., 2018). In addition, FFA is widely used for the preparation of cast resins via cross-linked polymerization with itself and other compounds, such as FF, formaldehyde, phenolic compounds, and urea. Besides showing corrosion and solvent resistance, the resulting resins have excellent chemical, thermal and mechanical properties (Barr and Wallon, 1971).

Similar to FFA, 2,5-bis(hydroxymethyl)-furan (BHMF) is derived from the hydrogenation of 5-hydroxymethylfurfural (HMF) that can be originated from hexose sugars such fructose, glucose, and cellulose by hydrolysis/dehydration (Román-Leshkov et al., 2006; Zhao et al., 2007; Hu et al., 2009; Qi et al., 2009; Chen et al., 2014a) and is known as a key platform molecule for the production of various valuable products, such as 2,5-dimethylfuran (DMF), 2,5-dihydroxymethyl-tetrahydrofuran (DHMTHF), 2,5-dimethoxytetrahydrofuran (DMTHF), and 5-methylfurfural (MF) (Román-Leshkov et al., 2007; Kwon et al., 2013; Audemar et al., 2014; Hu et al., 2014; Revunova and Nikonov, 2015; Seemala et al., 2017, 2018; Wang T. et al., 2017). As one of the hopeful bio-based downstream products, BHMF can be used to prepare a variety of functional materials (e.g., polymers, resins, crown ethers, and rayon) and as universal brackets for the preparation of drugs and related bioactive compounds (Han et al., 2016).

**Figure 2:**
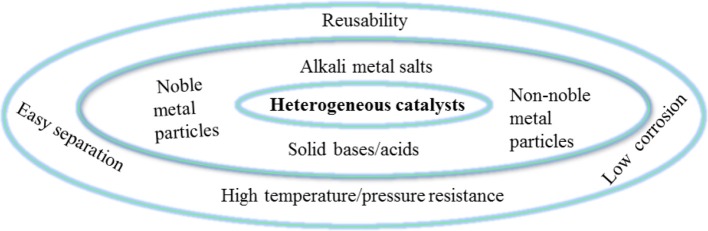
Advantages of heterogeneous catalysts.

Both homogeneous and heterogeneous catalysts can be used for biomass upgrading. The active site of the homogeneous catalyst is in the same phase as the reactant, and can effectively interact with the reaction substrate, usually resulting in a higher turnover frequency (TOF) rates than that of the heterogeneous catalyst. However, homogeneous catalysts are not widely used due to many disadvantages, such as high toxicity, corrosion, difficulty in separation and purification, and inefficient reusability (Sheldon, 2005). Heterogeneous catalysts are widely used in biomass upgrading reactions in view of their many advantages, as shown in Figure 2. For example, heterogeneous catalytic systems are less corrosive. Another advantage of heterogeneous catalysts is that they are highly stable to harsh reaction conditions (high temperature and pressure) (Kokel et al., 2017). What's more, heterogeneous solid catalysts can be easy to separate and highly reusable in continuous catalytic cycles, making the process cost-effective and more sustainable. Over the past several decades, different types of heterogeneous catalysts and advanced methods have been explored to catalyze the hydrogenation of bio-based furanic aldehydes to alcohols (Chen et al., 2014a). Several research groups are currently studying this reaction using various noble metals, such as Pd (Audemar et al., 2014; Li et al., 2018c), Pt (An et al., 2013; Shi et al., 2016), and Ru (Nishimura et al., 2014; Yuan et al., 2015b), non-noble metals, such as Cu (Lesiak et al., 2014; Upare et al., 2018) and ferrous metals (Fe, Ni, Co) (Yao et al., 2014; Yu et al., 2015; Halilu et al., 2016), solid acid-base catalysts, and alkali metal salt catalysts [e.g., K_2_CO_3_ (Long et al., 2018), Cs_2_CO_3_ (Long et al., 2019) and KF (Wu et al., 2018; Zhao et al., 2018)] for the catalytic upgrading of biomass-derived furanic aldehydes to alcohols.

Several important review articles have been reported on the development of heterogeneous catalysts for biomass upgrading reactions. For instance, Lin and Huber (2009) provided a review on the pivotal role of heterogeneous catalysis in biomass upgrading. In another review, Yan et al. (2014) have discussed heterogeneous catalysts for the hydrogenation of FF into fuel additives and value-added chemicals by advanced catalytic hydrogenation technologies. Gabriëls et al. (2015) reported the applications of homogeneous, heterogeneous, and combined homogeneous and heterogeneous catalytic systems in Guerbet chemistry. Gilkey and Xu (2016) provided a review on the upgrading of biomass-derived feedstocks through heterogeneous catalytic transfer hydrogenation (CTH), and discussed the challenges and opportunities of establishing CTH in the effective production of biofuels and renewable chemicals. Recently, Sudarsanam et al. (2018) provided a review on developing functional heterogeneous catalysts, such as carbon materials, metal-organic skeletons, solid-phase ionic liquids, and magnetic ferric oxide, to efficiently upgrade biomass. Herein, this review provides a critical review on catalytic upgrading of biomass-derived furanic aldehydes to alcohols by using various heterogeneous catalysts. The aim of this review is to provide a comprehensive overview of the production of BHMF or FFA from the hydrogenation of 5-hydroxymethylfurfural or FF. This review evaluates the recent progress of developing different heterogeneous catalytic materials, such as noble/non-noble metal particles, solid acids/bases, and alkali metal salts, for the efficient reduction of bio-based furanic aldehydes to alcohols. Emphasis is laid on the insights and challenges encountered in those biomass transformation processes.

## Catalytic Strategies Toward Furanic Platforms

**Scheme 1:**
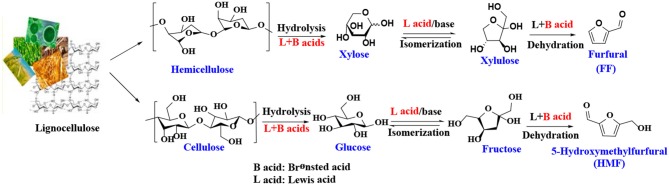
Schematic illustration of FF and 5-hydroxymethylfurfural production from biomass.

There have been many reports on the different reaction mechanisms for the production of FF from xylose (Marcotullio and De Jong, 2010; Yang et al., 2012), but most studies using heterogeneous catalysts are based on a cyclized dehydration mechanism that gradually releases three molecules of water (Marcotullio and De Jong, 2010). This mechanism typically uses Brønsted acid catalysts, while some modifications have been made to the Lewis acid catalysts to produce FF. As shown in Scheme 1, xylose is isomerized to xylulose in the presence of Lewis acid or base sites, which has been confirmed by several experimental studies (Takagaki et al., 2010; Choudhary et al., 2011; Tuteja et al., 2012). For example, Choudhary et al. (2011) reported that Lewis acid zeolite primarily converts xylose to xylulose with almost no FF formed, while in the presence of Brønsted acid catalysts, such as HCl and Amberlyst-15, the intermediate xylulose can be rapidly converted to FF. The properties of heterogeneous catalysts, such as acidity (acid type, strength, and amount), structure (pore size and surface area), hydrophobicity and stability, can significantly affect catalytic performance. For acidic species, the Lewis acid site can transfer the reaction pathway to the xylose-xylulose-FF route, which is faster than the direct reaction of xylose to the FF route by the Brønsted acid sites. In the presence of both Brønsted and Lewis acid sites, it is faster to catalyze the dehydration of xylulose to produce FF and acquire a higher yield of FF (Li et al., 2016d). Similar to the formation mechanism of FF, 5-hydroxymethylfurfural is obtained by dehydration of glucose or fructose or by hydrolysis of cellulose in the presence of both Lewis and Brønsted acid catalysts (Scheme 1) (Zakrzewska et al., 2011; Cai et al., 2014b; Svenningsen et al., 2018).

## Catalytic Hydrogenation of Biofuranic Aldehydes to Alcohols

### Non-precious Metal Particles

#### Cu-Based Catalysts

Hydrogenation of FF to FFA is usually carried out in a gas phase or a liquid phase process. Compared to gas phase hydrogenation, gas phase hydrogenation is generally preferred because it can be carried out under normal pressures. The gas phase catalytic process was first reported in 1929 by the use of copper supported as a catalyst in asbestos (Ricard and Guinot, 1929). Copper chromite catalysts have been widely used in various industrial processes, such as the decomposition or dehydration of the alcohol and the partial hydrogenation of vegetable oils and fatty acids (Rao et al., 1997). However, the main disadvantage of Cu-Cr catalysts is their high toxicity (Li et al., 2006). The final disposal of the catalyst causes serious environmental pollution (Huang et al., 2007). In order to decrease the chromium content of the Cr-containing catalyst while showing comparable catalytic activity, Huang et al. studied the application of TiO_2_ supported copper chromite catalyst in the selective hydrogenation of FFA, and the conversion of FF was 100% and the yield of FFA was 79% under the reaction conditions of 140°C for 2 h (Huang et al., 2007). The Cu-Cr catalyst is industrially used for the hydrogenation of FF at high temperature and pressure to produce FFA with moderate activity and selectivity (Baijun et al., 1998; Wu et al., 2005; Li et al., 2006). Burch et al. (1990) reported that ZnO had an important supporting role and could act as a reservoir of atomic hydrogen to promote hydrogen overflow in the reaction. It is known that the role of ZnO is to increase the dispersion of Cu, which can improve the performance of the catalyst (Fei et al., 2004; Pillai and Deevi, 2006). Therefore, in order to increase the conversion and selectivity of FF hydrogenation to FFA, Zn is added to the Cu(3):Cr(1) catalyst. Zirconium is known to enhance the acidity of the catalyst and also to increase the selectivity of the hydrogenation reaction (Pillai and Deevi, 2006; Liu et al., 2012). Zr was thus added into the Cu(3):Zn(2):Cr(1) catalyst to increase the selectivity of FFA formation. The results showed that Cu:Zn:Cr:Zr (3:2:1:3) was the optimal catalyst for the reduction of FF to FFA. Under the reaction conditions of 170°C and 3.5 h, the conversion of FF was 100% and selectivity reached 96% (Sharma et al., 2013).

The high toxicity of the chromium-containing catalysts promotes the development of new Cr-free catalytic systems. New restrictions now prevent the used chromite catalysts from being deposited in landfills, providing the impetus for the exploration of new alternative Cu catalysts free of chromium. Therefore, many non-chromium catalysts have been developed for the hydrogenation of FF and 5-hydroxymethylfurfural in the gas phase and liquid phase in the presence of different catalysts, as summarized in Table 1. To date, various single metal and bimetallic catalysts have been prepared, which are supported on relatively inert materials, such as silica and alumina. Among Cu-based catalysts, the main reason for using promoters and developing the multi-metallic catalysts was the utilization of a multi-component catalyst system to improve the catalyst performance, comparing to the single metal counterparts. Wang Y. et al. (2017) reported the conversion of FF was 98.7% and selectivity reached 96.4% by the catalyst CuCo0.4/C-873, but the selectivity of FFA and the conversion of FF were only 81.3 and 46.6%, respectively, with Cu/C-873 catalyst, indicating the promoting effect of cobalt. The increase in furfuryl alcohol selectivity may be due to the doping of cobalt resulting in better dispersion of the metal particles, which allows for good regulation of the structure of the active site. Selectively activating the C = O bond of the FF and the decomposition of hydrogen is a necessary condition for the hydrogenation of FF to furfuryl alcohol. In the catalytic system of Cu/C-873, metallic copper acts as an active site for activating the C = O bond and dissociating hydrogen molecules. However, copper with relatively large particle size is less active during hydrogen activation (Takagaki et al., 2010; Vargas-Hernández et al., 2014). By doping with cobalt, smaller and more easily dispersible copper and cobalt particle sizes can be found in the bimetallic CuCo/C-873 catalyst, which will contribute to generating more active reactive sites (Li et al., 2016d). Due to the presence of cationic species, in addition, the interaction of cobalt oxide (CoOx) with the C = O group of FF is more intense (Rao et al., 1997). Therefore, more CoOx sites can be obtained in a catalyst with high cobalt content and used to activate more C = O groups. In addition, metallic cobalt is more active than copper in terms of hydrogen dissociation. Although the metal cobalt alone exists, the C = O bond and the furan ring can be activated simultaneously. In contrast, the synergistic action of copper and cobalt prevents the activation of the furan ring, thus exhibiting the pronounced selectivity toward the partially hydrogenated product. Therefore, the catalytic performance of the bimetallic CuCo/C-873 catalyst is better than that of Cu/C-873 (Wang Y. et al., 2017). Unfortunately, the bimetallic CuCo/C-873 catalyst has a significant loss in catalytic activity over four consecutive cycles. Similarly, Chen et al. reported 76.8% BHMF selectivity and 70% 5-hydroxymethylfurfural conversion over catalyst Cu/C but only 82.9% BHMF selectivity and 63.9% conversion over CuZn/C in aqueous hydrogenation of 5-hydroxymethylfurfural to BHMF, illustrating the promoting effect of zinc (Chen et al., 2017). In addition to the above metals, the doping of metals, such as Ni, Fe, Mg, Al, etc. also improves the activity, selectivity, and the stability of copper-based catalysts for liquid-phase catalytic reactions (Villaverde et al., 2013; Yan et al., 2013b; Khromova et al., 2016).

**Table 1:** Hydrogenation of FF and 5-hydroxymethylfurfural catalyzed by different non-precious metallic catalysts.

**Substrate**	**Catalyst**	**Reaction condition**			**H-donor**	**Main product**	**Catalytic activity**			**Reusability**				**References**
		**Solvent**	**Temp. (°C)**	**Time (h)**			**Conv. (%)**	**Yield (%)**	**Selec. (%)**	**Cycles**	**Yield (%)**	**Selec. (%)**	**Conv. (%)**	
FF	CuCo0.4/C-873	Ethanol	140	1	3 MPa H_2_	FFA	98.7	95.1	96.4	Four	69	90	77	Wang Y. et al., 2017
FF	Cu/C-873	Ethanol	140	1	3 MPa H_2_	FFA	46.6	37.9	81.3	NM	NM	NM	NM	Wang Y. et al., 2017
HMF	Cu/C	Ethanol	180	8	5 MPa H_2_	BHMF	70	54	76.8	NM	NM	NM	NM	Chen et al., 2017
HMF	CuZn/C	Ethanol	180	8	5 MPa H_2_	BHMF	63.9	53	82.9	NM	NM	NM	NM	Chen et al., 2017
FF	Ni–Cu	Decyl alcohol	130	2	5 MPa H_2_	FFA	-	-	100	NM	NM	NM	NM	Khromova et al., 2016
FF	Cu–Fe	Octane	160	5	9 MPa H_2_	FFA	91	89.5	98	NM	NM	NM	NM	Yan et al., 2013b
FF	Cu–Fe	Octane	220	5	9 MPa H_2_	FFA	99.2	57.9	58	Three	70	78	90	Yan et al., 2013b
FF	CuMgAl	Isopropanol	350	1.5	1 MPa H_2_	FFA	100	100	100	NM	NM	NM	NM	Villaverde et al., 2013
HMF	Cu(50)-SiO_2_	BuOH	100	4	1.5 MPa H_2_	BHMF	95	93	98	Five	93	98	95	Upare et al., 2018
HMF	Cu/ZnO	1,4-dioxane	100	2	1.5 MPa H_2_	BHMF	100	99.1	99	NM	NM	NM	NM	Zhu et al., 2015
HMF	Cu/PMO	Ethanol	100	3	5 MPa H_2_	BHMF	100	99	99	Eight	86	92	93	Kumalaputri et al., 2014
HMF	CuNi/Al_2_ O_3_(Cu/Ni = 1)	Tetrahydrofuran	130	6	3 MPa H_2_	BHMF	70.6	62.4	88	NM	NM	NM	NM	Srivastava et al., 2017
FF	Cu-Ni/γ-Al_2_O_3_(Cu/Ni = 1)	Isopropanol	130	4	4 MPa H_2_	FFA	92.6	87	93.6	Four	32	32	100	Srivastava et al., 2017
FF	CM-Co(Cu-MgO)	Gas phase	200	6	In a flow of 6% H_2_ in He mixture	FFA	93	93	100	NM	NM	NM	NM	Nagaraja et al., 2011
FF	CuZnO-0.2	CPME	190	24	H_2_ flow = 10 ml·min^−1^	FFA	60	59	98	NM	NM	NM	NM	Jiménez-Gómez et al., 2016
FF	Cu_11.2_Ni_7.4_-MgAlO	Ethanol	300	2	1 MPa H_2_	FFA	93.2	89.2	96	NM	NM	NM	NM	Xu et al., 2011
FF	CoCu-MgO/CaCu-MgO	WHSV of 1.7 1/h	180	4	0.1 MPa H_2_	FFA	91	90	99	NM	NM	NM	NM	Ghashghaee et al., 2017
FF	CuNi/MgAlO	Ethanol	100	4	4 MPa H_2_	FFA	>99	99	100	Six	99	100	99	Wu et al., 2017
FF	Cu/TiO_2_	CPME	125	4	1 MPa H_2_	FFA	100	99	99	Three	94	99	95	Romano et al., 2016
FF	Cu/Al_2_O_3_	Gas phase	90	2	2 MPa H_2_	FFA	81	81	100	NM	NM	NM	NM	Lesiak et al., 2014
FF	Cu/γ-Al_2_ O_3_(10)	Isopropanol	130	4	4 MPa H_2_	FFA	64.2	47	72.6	NM	NM	NM	NM	Srivastava et al., 2017
FF	SBA-15Cu(SiO_2_)	Gas phase	230	1/4	H_2_ flow = 10 mL/min	FFA	91.5	85.4	93.1	NM	NM	NM	NM	Vargas-Hernández et al., 2014
HMF	Cu/AlOx	1,4-butanediol	220	CF	1,4-butanediol	BHMF	94	93	99	NM	NM	NM	NM	Aellig et al., 2014
HMF	Cu/γ-Al_2_O _3_(10)	Tetrahydrofuran	130	6	3 MPa H_2_	BHMF	55	40	73	NM	NM	NM	NM	Srivastava et al., 2017
HMF	Ni/γ-Al_2_O_3_(10)	Tetrahydrofuran	130	6	3 MPa H_2_	BHMF	15	7	45	NM	NM	NM	NM	Srivastava et al., 2017
FF	Ni/γ-Al_2_O_3_(10)	Tetrahydrofuran	130	4	4 MPa H_2_	FFA	22.6	20	91.6	NM	NM	NM	NM	Srivastava et al., 2017
FF	Ni/NAC-1-1073	Isopropanol	140	5 h	4 MPa H_2_	FFA	>99	99	100	Five	17	50	33	Gong et al., 2017
FF	Ni/CN	Isopropanol	200°C	4	1 MPa H_2_	FFA	96	91	95	Four	91	95	96	Kotbagi et al., 2016
FF	Ni–Fe(2)HT-673	Isopropanol	150	3	3 MPa H_2_	FFA	99	95	96	NM	NM	NM	NM	Putro et al., 2017
FF	Ni-Fe-B	Ethanol	100	4	1 MPa H_2_	FFA	100	100	100	NM	NM	NM	NM	Li et al., 2003
HMF	NiFe/CNT	1-butanol	110	18	3 MPa H_2_	BHMF	100	96.1	96.1	NM	NM	NM	NM	Yu et al., 2015
FF	Fe(NiFe)O_4_-SiO_2_	Heptane	250	4	0.5 MPa H_2_	FFA	93.5	93.5	100	NM	NM	NM	NM	Halilu et al., 2016
FF	Ni-Sn(1.0)/AlOH	Isopropanol	180	5/4	3 MPa H_2_	FFA	97	97	100	NM	NM	NM	NM	Rudiansono et al., 2014
FF	NiCoB	Ethanol	80	3	2 MPa H_2_	FFA	46	42	91	NM	NM	NM	NM	Du, 2011
FF	Fe-L1/C-800	2-butanol	160	6	2-butanol	FFA	71.3	60	84.2	Five	60	85	74	Li et al., 2016c
HMF	CoAl	Methanol	120	4	4 MPa H_2_	BHMF	89.4	83	93	NM	NM	NM	NM	Yao et al., 2014
HMF	CoxOy	Methanol	90	1	2 MPa H_2_	BHMF	–	93	–	NM	NM	NM	NM	Li et al., 2018d
HMF	Co_3_O_4_ @MC	Isopropanol	140	12	Isopropanol	BHMF	100	97	97	NM	NM	NM	NM	Wang G. H. et al., 2016

*CPME, Cyclopentyl methyl ether; WHSV, Weight hourly space velocity; NM, Not mentioned; CF, Continuous flow*.

The addition of metal or non-metal oxides is also beneficial to increase the catalytic activity and stability of Cu-based catalysts. For example, Nagaraja et al. (2011) used the Cu-MgO-Co to catalyze the hydrogenation of FF, and the conversion and selectivity of FFA reached more than 90% at 225°C after 6 h. This may result from the higher surface and smaller crystallite size on the surface of the support. (Jiménez-Gómez et al., 2016) also reported that the use of amphoteric oxides, such as ZnO could change the electron density and dispersion of metallic copper, which can improve catalytic activity and resistance to deactivation (Dong et al., 2015). In the presence of Cu-ZnO, the conversion of FF and the selectivity of FFA were 93 and 82%, respectively (Jiménez-Gómez et al., 2016). Xu et al. reported that Cu was added to the hydrotalcite precursor layer by coprecipitation method, and the solid was calcined to prepare a Cr-free Cu-based catalyst, namely Cu_11.2_Ni_4.7_-MgAlO, in which the highly dispersed metal particles can well-contact with hydrogen to promote FF hydrogenation. Gratifyingly, this catalytic system gave FF conversion and FFA selectivity of 93.2 and 89.2% at 300°C, respectively (Xu et al., 2011). Ghashghaee et al. (2017) reported a novel method based on a combination of coprecipitation and hydrothermal methods for the preparation of a series of Cu-MgO catalysts containing various promoters (Co and Ca) and certain morphology. Ca is a basic promoter, and an appropriate amount of Ca can improve the thermal stability of the Cu-based catalyst in the hydrogenation reaction (Burch et al., 1990). According to previous reports, Co promoters can increase the selectivity of the reaction process by decreasing unwanted by-products (Reddy et al., 2007). Under the positive influence of two promoters (Co and Ca), the selectivity of FFA was over 99% (Ghashghaee et al., 2017). In addition to other metals, such as nickel, Cobalt, iron and magnesium species, the selectivity of FFA was relatively high (Villaverde et al., 2013; Yan et al., 2013b; Khromova et al., 2016; Wang Y. et al., 2017; Wu et al., 2017). The Cu supported on relatively inert materials, such as silica, ceria, zinc oxide, and alumina were capable of catalyzing the hydrogenation of FF to FFA, showing good performance with respect to the conversion of FF and the selectivity toward FFA (Sitthisa et al., 2011a; Lesiak et al., 2014; Vargas-Hernández et al., 2014; Srivastava et al., 2015; Romano et al., 2016; Du et al., 2018; Jiménez-Gómez et al., 2018; Yang et al., 2018).

Upare et al. (2018) reported the production of BHMF from 5-hydroxymethylfurfural using Cu(50)-SiO_2_, which were prepared with different Cu loading by precipitation-deposition method, achieving a BHMF yield of 93% and a 95% conversion of 5-hydroxymethylfurfural in BuOH at 100°C for 4 h under 1.5 MPa H_2_. The catalyst could be reused for five cycles with consistent activity. (Zhu et al., 2015) later utilized a copper-zinc oxide to catalyze the hydrogenation of 5-hydroxymethylfurfural to BHMF, achieving a 99.1% yield and a 100% 5-hydroxymethylfurfural conversion at 100°C for 2 h in presence of 1.5 MPa H_2_. For catalytic hydrogenation of 5-hydroxymethylfurfural, Cu-ZnO catalyst has high activity and selectivity, which can be attributed to high dispersion of Cu particles produced by strong interaction between copper and zinc oxide, and synergistic effect between surface sites of metal copper and weakly acidic sites from zinc oxide. (Kumalaputri et al., 2014) utilized a hydrotalcite-derived copper catalyst to catalyze the hydrogenation of 5-hydroxymethylfurfural, giving a BHMF yield of 99% and a 5-hydroxymethylfurfural conversion of 100%. Unfortunately, because the carbon species deposited on the catalyst surface, the catalyst activity was maintained for 3 cycles, and then the BHMF selectivity gradually decreased to 61, 53, 38, and 19% in the fourth to seventh cycles, respectively. Aellig et al. (2014) reported the AlO_*x*_ supported Cu catalyst, which was synthesized by direct reduction of Cu-Al hydrotalcite precursor, for the hydrogenation of 5-hydroxymethylfurfural to BHMF. The AlO_*x*_ supported copper showed better catalytic performance as compared with aluminum oxide supported one (Srivastava et al., 2017), achieving a 93% BHMF yield and 100% 5-hydroxymethylfurfural conversion at 100°C by using 1,4-butanediol as H-donor, demonstrating that the reduction temperature of the catalyst had an important effect on the catalytic activity, and a higher reduction temperature resulted in higher activity but the catalyst was less stable (Aellig et al., 2014).

**Scheme 2:**
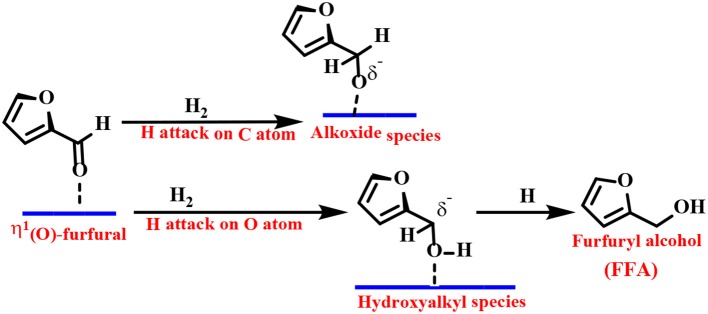
Mechanism of FF hydrogenation to furfuryl alcohol (FFA) over a Cu-based catalyst.

Density functional theory (DFT) calculations and infrared (IR) spectroscopy techniques have been employed to provide explanations for the adsorbent patterns of different intermediates and reasonable reaction pathways. As one of the representative examples, Resasco's group proposed a Cu-based catalyst to catalyze the reduction of FF to FFA. They proposed that the adsorption of FF preferentially passes through the η^1^(O)-aldehyde binding mode, as shown in Scheme 2 (Sitthisa et al., 2011b). The FF molecule is perpendicular to the surface of the catalyst, and the aromatic ring undergoes a net repulsion due to the overlap of the three-dimensional band of the surface Cu atoms with the aromatic furan ring. Thus, the reaction can be carried out by an alkoxide (H attack on the C atom of the carbonyl group) or a hydroxyalkyl group (H addition to the O atom of the carbonyl group) intermediate. The latter mechanism is preferred because it has a lower activation energy barrier, which can be explained by the stabilizing effect of the aromatic furan ring on the hydroxyalkyl intermediate (Chen et al., 2014b).

#### Ferrous Metal (Fe, Ni, Co)-Based Catalysts

Among non-precious metals, metallic nickel is distinguished for its remarkable hydrogenation capacity. However, there are very few applications in the reduction of 5-hydroxymethylfurfural and FF to BHMF or furfuryl alcohol because of the strong interaction between the furan ring and the Ni site, resulting in the easy opening of the furan ring, which reduces the selectivity of BHMF and FFA. Merat et al. (1990) reported the use of a nickel catalyst to catalyze the hydrogenation of FF, and the main product was FFA at 130°C. The selective reduction ability of the single metal Ni catalysts in the reduction of FF to FFA is relatively poor because they have too high hydrogenation activity. Therefore, it is desirable to modify the nickel to increase the selective reduction of the aldehyde group to escape the occurrence of undesirable side reactions. The following three methods are a large number of previous studies: (i) using oxides, such as Al_2_O_3_ (Srivastava et al., 2017), SiO_2_ (Halilu et al., 2016) or *N*-doped activated carbon supports to interact strongly with nickel (Gong et al., 2017), (ii) adding oxyphilic metals, such as Fe (Li et al., 2003), Sn (Rudiansono et al., 2014), and Co (Du, 2011), and (iii) controlling the reaction conditions to restrain the opening reaction of the furan ring. For example, Srivastava et al. (2017) reported that Ni-loaded alumina was used to catalyze the hydrogenation of 5-hydroxymethylfurfural and FF with undesirable effect, resulting only into 15 and 22.6% conversion of 5-hydroxymethylfurfural and FF, respectively. Gong et al. (2017) used doped activated carbon supported metallic nickel (Ni/NAC), which was prepared by self-assembly in two-step calcination in nitrogen, to catalyze the hydrogenation of FF to FFA, giving excellent performance at 140°C for 2 h in the presence of 4 MPa H_2_. After consecutive five cycles, the catalytic activity of the catalyst was significantly reduced, and the conversion of FF was reduced from 99 to 33%. Kotbagi et al. (2016) reported that Ni/CN monolithic catalysts with different Ni content, which were prepared by using a one-pot co-gelation sol-gel technique, catalyzed the conversion of FF to FFA. 96% FF conversion and 95% selectivity FFA were obtained at 200°C for 4 h under 1 MPa H_2_. After the catalyst was reused four times, the reactivity did not decrease, indicating the preparation method of the catalyst has a great influence on the stability of the catalyst. Merging a third constituent, such as Fe, Sn, or Co, improves the catalytic activity. Iron is widely known for its abundant reserves, low price, environmental friendliness, and ease of recycling. Fe is mainly utilized as an oxophilic metal additive to enhance the reduction performance of other metals, such as copper (Yan et al., 2013b). Most importantly, the existence of oxophilic Fe as a doped additive improves the specific surface area of the catalyst, while the dosage of unsaturated Ni active sites and the distribution of Ni active sites facilitates the adsorption of C = O bonds of FF. Putro et al. (2017) adopted a simple hydrothermal method to prepare Ni-Fe alloy catalyst, and used to catalyze the reduction of FF to FFA, acquired excellent performance with a 99% FF conversion and a 95% FFA yield at 150°C for 3 h under a hydrogen pressure of 3 MPa. In order to increase the stability of the Ni-Fe catalyst, some researchers have added some supporters to the catalyst. For example, Li et al. (2003) reported the reduction of FF to FFA over the Fe-promoted Ni-B amorphous alloy catalysts (Ni-Fe-B), which was prepared by reducing mixed FeCl_3_ and NiCl_2_ with KBH_4_ in aqueous solution, in the liquid phase. The yield of FFA was 100% with complete conversion of FF, revealing that the strong interaction between boron and nickel accelerated the activation of the C = O bond in FF, and thereby promoted the selective formation of FFA. 5-Hydroxymethylfurfural could be selectively hydrogenated with carbon nanotube-supported bimetallic Ni-Fe (Ni-Fe/CNT) catalysts as shown by (Yu et al., 2015). A 96.1% BHMF yield and complete 5-hydroxymethylfurfural conversion were obtained under the conditions of 110°C and 3 MPa H_2_ for 18 h (Yu et al., 2015). Magnetic catalysts are well-known for facilitating catalyst separation with excellent catalytic activity, which is favored by many researchers. Halilu et al. (2016) reported magnetite Fe(NiFe)O_4_-SiO_2_ nanoparticles, which were synthesized by a facile co-precipitation method, catalyzed the hydrogenation of FF to FFA. Satisfactorily, this catalytic system gave FF conversion and FFA selectivity of 93.5% at 250°C for 2 h under hydrogen pressure of 0.5 MPa. Incorporating another third component, such as Sn (Rudiansono et al., 2014) or Co (Du, 2011) also increases the catalytic performances. Hydrogenation with hydrogen as a source of hydrogen has certain weaknesses, and high-pressure hydrogen often poses a safety hazard (He et al., 2018b; Long et al., 2019). Catalytic transfer hydrogenation (CTH) can be used as an alternative to reduce biomass-derived molecules, and a hydrogen donor (e.g., secondary alcohol) is used as a hydrogen source instead of molecular H_2_. Monometallic iron-based catalysts only exhibited low to moderate catalytic reactivity in the hydrogenation of FF. But several latest reports showed that this could be obviously promoted by C or N supporters (Li et al., 2003). Li et al. (2016c) utilized nitrogen-doped carbon-supported iron catalysts, which were prepared through simultaneous pyrolysis of metal complexes and carbon materials, for the hydrogenation of FF to furfuryl alcohol using 2-butanol as H-donor. A moderate FF conversion of 73.3% and FFA yield of 60% was obtained at 250°C. The catalyst was able to be reused for 5 times with no significant loss of catalytic activity. In addition to the above non-precious catalysts, cobalt-based catalysts were also utilized to catalyze the hydrogenation of FF and 5-hydroxymethylfurfural, giving FFA and BHMF in good yields (83–97%) (Yao et al., 2014; Wang G. H. et al., 2016; Li et al., 2018d).

### Precious Metal Particles

Various noble metals and non-noble metals have been reported for the selective hydrogenation of FF and 5-hydroxymethylfurfural in the gas phase and the liquid phase. Liquid phase hydrogenation was investigated using different chromium and non-chromium catalysts (Mariscal et al., 2016). Generally, the Cu-based catalyst exhibits a remarkable selectivity for the hydrogenation of a carbonyl group, and the C-C double bond in the furan ring does not participate in the reaction. A second metal or promoter is sometimes added to improve activity or selectivity by increasing the surface area or acting as a Lewis acid site to polarize the C-O bond. However, the main drawback is that most of these catalysts cannot be reused (Wu et al., 2005). Therefore, other noble metals, such as Pd (Lesiak et al., 2014), Pt (Huang et al., 2016), Ru (Han et al., 2016), Ir (Nakagawa et al., 2014), and Au (Li et al., 2015), were added to increase their stability, where ethanol, water, ethanol, isopropyl alcohol, *n*-butyl alcohol, benzyl alcohol, heptane, tetrahydrofuran, or octane was used as the solvent, as shown in Table 2.

**Table 2:** Reduction of FF and 5-hydroxymethylfurfural catalyzed by different precious metallic catalysts.

**Substrate**	**Catalyst**	**Reaction condition**			**H-donor**	**Main product**	**Catalytic activity**			**Reusability**				**References**
		**Solvent**	**Temp. (°C)**	**Time (h)**			**Conv. (%)**	**Yield (%)**	**Sele. (%)**	**Cycles**	**Yield (%)**	**Sele. (%)**	**Conv. (%)**	
FF	Pd/CB	Ethanol	50	2	2 MPa H_2_	FFA	48	47	98	NM	NM	NM	NM	Mironenko et al., 2015
FF	Pd/SBA-15	Gas phase	150	1/3	0.3 mL min^−1^	FFA	40	40	100	NM	NM	NM	NM	Ouyang et al., 2018
FF	Pd/Al_2_O_3_	Octane	150	6	4.5 MPa H_2_	FFA	21.2	19	90.3	NM	NM	NM	NM	Yan et al., 2013a
FF	Pd/Al_2_(SiO_3_)_3_	Toluene	150	4	0.2 MPa H_2_	FFA	56.9	30	53	NM	NM	NM	NM	Yu et al., 2011
FF	Pd_2_AuHMS	Isopropanol	220	5	3.4 MPa H_2_	FFA	42	36	86	NM	NM	NM	NM	Date et al., 2018
FF	HF-treated Pd@S-1 w	n-butanol	250	2	10% H_2_/Ar at a rate of 10 mL/min,	FFA	81	33	40.1	NM	NM	NM	NM	Wang C. et al., 2016
FF	Pd/Al_2_O_3_/Ru/ZrO_2_	Water	30	3	0.5 MPa H_2_	FFA	28	27	97	NM	NM	NM	NM	Huang et al., 2018
FF	Pd-10Cu/C	Water	80	1.5	0.6 MPa H_2_	FFA	96.6	86	89.1	NM	NM	NM	NM	Fulajtárova et al., 2015
FF	Pd-Cu/MgO	Water	130	0.9	0.6 MPa H_2_	FFA	100	99	99	Four	80	82	98	Fulajtárova et al., 2015
FF	Pd-Cu/Al_2_O_3_	Aqueous solution	90	2	2 MPa H_2_	FFA	–	–	100	NM	NM	NM	NM	Lesiak et al., 2014
FF	Pd/Al_2_O_3_/Ru/ZrO_2_	Water	30	3	0.5 MPa H_2_	FFA	28	27	97	NM	NM	NM	NM	Huang et al., 2018
FF	Pd/TiO_2_	Octane	25	2	0.3 MPa H_2_	FFA	65.4	28.6	44	NM	NM	NM	NM	Aldosari et al., 2016
FF	Pd/Al_2_(SiO_3_)_3_	Toluene	150	4	0.2 MPa H_2_	FFA	56.9	30	53	NM	NM	NM	NM	Yu et al., 2011
FF	Pd/Fe_2_O_3_	Isopropanol	180	7.5	Isopropanol	FFA	87	57	66	NM	NM	NM	NM	Addis et al., 2011
FF	Pd/C-wet	n-butanol	100	2	PMHS	FFA	99	95	96	NM	NM	NM	NM	Li et al., 2018c
HMF	Pd/C	Tetrahydrofuran	80	20	10 MPa H_2_	BHMF	97	82	85	NM	NM	NM	NM	Audemar et al., 2014
FF	Pt-Cu nanoparticles	Methanol	150	24	2.0 MPa H_2_	FFA	40	100	100	NM	NM	NM	NM	Huang et al., 2016
FF	Pt(3)Co(3)/C	Isopropanol	100	5	1 MPa H_2_	FFA	100	100	100	Three	100	100	100	Dohade and Dhepe, 2017
FF	Pt-g-C_3_N 45%(Pt@TECN)	Water	100	5	1 MPa H_2_	FFA	>99	>99	>99	NM	NM	NM	NM	Chen et al., 2016
FF	Pt-NPs@P-SiO_2_	Heptane	80	4	4 MPa H_2_	FFA	100	100	100	NM	NM	NM	NM	Castelbou et al., 2017
FF	Pt/γ-Al_2_O_3_	Methanol	50	7	0.1 MPa H_2_	FFA	80	79.2	99	NM	NM	NM	NM	Taylor et al., 2016
HMF	Pt/C	Ethanol	23	18	1.4 MPa H_2_	BHMF	–	82	–	NM	NM	NM	NM	Balakrishnan et al., 2012
HMF	Pt/Al_2_O_3_	Ethanol	23	18	1.4 MPa H_2_	BHMF	–	85	–	NM	NM	NM	NM	Balakrishnan et al., 2012
HMF	Pt/C	Ethanol	60	5	1.4 MPa H2	BHMF	–	17	–	NM	NM	NM	NM	Balakrishnan et al., 2012
HMF	Pt/Al_2_O_3_	Ethanol	60	5	1.4 MPa H2	BHMF	–	45	–	NM	NM	NM	NM	Balakrishnan et al., 2012
HMF	Pt1Sn1/Al_2_O_3_	Ethanol	60	5	1.4 MPa H_2_	BHMF	–	82	–	NM	NM	NM	NM	Balakrishnan et al., 2012
HMF	PtSn/SnO_2_/RGO	Ethanol	70	0.5	2 MPa H_2_	BHMF	99	99	100	NM	NM	NM	NM	Shi et al., 2015
HMF	PtCo/HCS	n-butanol	120	2	1 MPa H_2_	BHMF	100	70	70	NM	NM	NM	NM	Wang et al., 2014
HMF	Pt/MCM-41	Water	35	2	0.8 MPa H_2_	BHMF	100	99	99	Six	98	–	–	Chatterjee et al., 2014
FF	Ru/Zr-MOFs	Water	20	4	0.5 MPa H_2_	FFA	–	94.9	–	Five	–	–	82	Yuan et al., 2015b
HMF	Ru/Cu Nanoparticles	Isopropanol	210	12	Isopropanol	BHMF	99	91.5	92	Third	90	93	97	Zhang et al., 2019
HMF	Ru/C	Ethanol	150	3	0.4 MPa H_2_	BHMF	–	95	–	NM	NM	NM	NM	Gupta and Saha, 2017
HMF	RuCo/C	Benzyl alcohol	150	10	Benzyl alcohol	BHMF	90.7	86.9	96	Four		75		Gao et al., 2016
HMF	Ru/Co_3_O_4_	Isopropanol	190	6	Isopropanol	BHMF	100	82.8	83	NM	NM	NM	NM	Wang T. et al., 2017
HMF	Ru/Al_2_O_3_	n-butanol-water	130	2	2.7 MPa H_2_	BHMF	92	74.5	81	NM	NM	NM	NM	Alamillo et al., 2012
HMF	Ru(OH)*_*x*_*/ZrO_2_	n-butanol	120	6	1.5 MPa H_2_	BHMF	99	99	100	Five	98	98	100	Han et al., 2016
HMF	Ru/ZrO_2_-SiO_2_	Water	25	4	0.5 MPa H_2_	BHMF	98.1	90.4	92	NM	NM	NM	NM	Chen et al., 2013
HMF	Ru/ZrO_2_-MgO	n-butanol-water	130	2	2.7 MPa H_2_	BHMF	99	93.1	94	NM	NM	NM	NM	Alamillo et al., 2012
HMF	Cm*Ru(HTsDPEN)	Tetrahydrofuran	40	2	HCOOH	BHMF	100	99	99	NM	NM	NM	NM	Thananatthanachon and Rauchfuss, 2010
FF	Ir-ReO*_*x*_*/SiO_2_	Water	30	6	0.8 MPa H_2_	FFA	>99	>99	100	NM	NM	NM	NM	Tamura et al., 2013
HMF	Ir-ReO*_*x*_*/SiO_2_	Water	30	6	0.8 MPa H_2_	BHMF	>99	>99	100	NM	NM	NM	NM	Tamura et al., 2013
FF	Ir@CN	Water	100	18	HCOOH	FFA	–	99	–	NM	NM	NM	NM	Wang et al., 2015
HMF	Cp*Ir(HTsDPEN)	Tetrahydrofuran	40	2	HCOOH	BHMF	100	99	99	NM	NM	NM	NM	Thananatthanachon and Rauchfuss, 2010
HMF	Cp*Ir(HTsDACH)	Tetrahydrofuran	40	1	HCOOH	BHMF	100	99	99	NM	NM	NM	NM	Thananatthanachon and Rauchfuss, 2010
HMF	Cp*Ir(NHCPh_2_C_6_H_4_)	Tetrahydrofuran	40	1	HCOOH	BHMF	100	99	99	NM	NM	NM	NM	Thananatthanachon and Rauchfuss, 2010
HMF	Cp*Ir(pyridinesulfonamide)Cl	Isopropanol	85	0.5	Isopropanol	BHMF	–	100	–	NM	NM	NM	NM	Townsend et al., 2017
FF	Au/ZrO_2_	Isopropanol	120	3	Isopropanol	FFA	100	100	100	NM	NM	NM	NM	Zhu et al., 2016
HMF	Au/Al_2_O_3_	Water	120	2	6.5 MPa H_2_	BHMF	>96	>96	100	NM	NM	NM	NM	Ohyama et al., 2016
HMF	Au/FeO*_*x*_*/Al_2_O_3_	Water	80	2	3 MPa H_2_	BHMF	96	96	100	NM	NM	NM	NM	Ohyama et al., 2016

Solvent properties (e.g., polarity) were also found to affect the interaction of the solvent with the reactants. Typically, with the increase of the polarity of the solvent, the interaction of FF with the solvent also increases. Accordingly, the yield of FFA would decrease as the adsorption of FF onto the catalyst was difficult. Among these solvents, water is the most polar solvent and should be the best medium for the reaction. Also, the product is easy to separate in this environment.

#### Pd-Based Catalysts

Palladium is well-known for its excellent catalytic performance in hydrogenation. Recently, different monometallic Pd-based materials were deeply studied for the hydrogenation of FF and 5-hydroxymethylfurfural. Palladium nanoparticles were deposited onto various supports, such as carbon (Audemar et al., 2014; Mironenko et al., 2015), SBA-15 silica (Ouyang et al., 2018), alumina (Yan et al., 2013a), titania (Aldosari et al., 2016), Al_2_(SiO_3_)_3_ (Yu et al., 2011), hexagonal mesoporous silica (Date et al., 2018), magnesium oxide (Fulajtárova et al., 2015), porous silicate (Biradar et al., 2013), and zeolite (Wang C. et al., 2016), showing good performance for the selective hydrogenation of FF and 5-hydroxymethylfurfural to FFA and BHMF, respectively. The improvement of C = O hydrogenation can be achieved by using a promoter, adjusting the particle size, changing the properties of the oxide support, and modifying the active metal with a second metal. The modification of the active metal with a second metal to form a bimetallic catalyst is an important factor in adjusting the catalytic selectivity of the C = O bond. The properties of bimetallic catalysts, such as electronic effects, synergistic effects, and alloy formation, significantly affect catalytic selectivity. In the Pd-based catalyst, the addition of a second metal, such as Cu (Fulajtárova et al., 2015) increased the hydrogenation selectivity of the various unsaturated carbonyl groups. In order to improve the performance of the catalyst, it is common to add a third metal. For example, Huang et al. (2018) added metal ruthenium to an alumina-supported palladium-based catalyst. Date et al. (2018) added metal Au to a palladium-based catalyst loaded with hexagonal mesoporous silica. In both cases, enhanced catalytic results were observed, as compared with the catalysts without doping additional metal species (Table 2, entry 5).

Although these catalytic systems have better catalytic effects, they all used hydrogen as a hydrogen source. To be noted, hydrogen is not easy to transport and store, making the experimental operation be cumbersome (Gandarias et al., 2012, 2013). In the presence of a catalyst, an alcohol is used as hydrogen donor, and FF is selectively converted to FFA by liquid-phase transfer hydrogenation (Yang et al., 2004; Nagaraja et al., 2007; Osatiashtiani et al., 2017), avoiding the use of inflammable hydrogen gas that involves complex storage and transportation process and thereby reducing the production cost of practical use. In this regard, it can solve the safety problems in the transportation process. In addition, non-corrosive alcohols can be obtained from sustainable biomass sources, and the dehydrogenation products (i.e., aldehydes or ketones) can be readily separated (Nagaraja et al., 2007; Bui et al., 2013; Jae et al., 2013). Therefore, the use of organic alcohols as hydrogen donors for the transfer hydrogenation of FF should be a more environmentally friendly alternative to conventional hydrogenation processes. Scholz et al. reported a Fe_2_O_3_-loaded Pd for the hydrogenation of FF to FFA with isopropanol as the hydrogen donor, with FFA yield and FF conversion in 57 and 87% at 180°C for 7.5 h, respectively. FFA cannot be selectively formed from FF in the presence of palladium, which on the other hand further undergoes hydrogenolysis to produce 2-methylfuran. Together with the hydrogenation reaction, FF undergoes decarbonylation to furan. As the conversion rate increased (the concentration of acetone increased accordingly), the aldol condensation product of FF and acetone 4-(furan-2-yl)but-3-en-2-one was detected. In addition, traces of furan-ring hydrogenated products, such as 2-methyltetrahydrofuran, (tetrahydrofuran-2-yl)methanol, and tetrahydrofuran were observed, indicating the high complexity of the reaction pathways (Addis et al., 2011). Unfortunately, the alcohol solvent can be further reacted with FFA by etherification to reduce its selectivity under the harsh conditions employed (Jae et al., 2013). Therefore, it is increasingly important to find an inexpensive and safe hydrogen donor. Polymethylhydrogensiloxane (PMHS), as a by-product of the silicon industry, is stable in water and air, and non-toxic and inexpensive (Addis et al., 2011). Li et al. (2018c) used PMHS as hydrogen source with Pd/C-wet to catalyze the hydrogenation of FF to FFA, giving FFA up to 95% yield at FF conversion of 99% under the reaction conditions of 15°C for 12 h. In addition to the formation of furfuryl alcohol, other substances, such as 2-methylfuran, 5-hydroxy-2-pentanone, and *n*-butyl levulinate were formed by appropriate control of the reaction parameters like reaction temperature and hydrosilane dosage.

#### Pt-Based Catalyst

Noble metal nanoparticles have proven to be effective catalysts in hydrogenation reactions (Durndell et al., 2015; Wei et al., 2015), which have been widely used in the industry. Among various noble metal nanoparticles, Pt catalyst is widely used in hydrogenation reactions due to its unique electrical and chemical properties (Chen et al., 2016). Due to complex side reactions (e.g., hydrogenolysis of C–O bonds, decarbonylation, over-hydrogenation and ring opening of furan), Pt-based catalysts are rarely used for the hydrogenation of FF and 5-hydroxymethylfurfural to FFA or BHMF (Chen et al., 2016). The physicochemical properties of the catalyst support significantly affect its liquid phase catalytic hydrogenation performance for unsaturated aldehydes and ketones (Mäki-Arvela et al., 2005). Pt-based catalysts have also been used in the FF or 5-hydroxymethylfurfural hydrogenation reaction by the addition of a suitable supporter. For example, Huang et al. (2016) using Pt-Cu nanoparticles catalyzed the hydrogenation of FF, giving the complete conversion of FF and 100% yield of FFA under the reaction conditions of 2 MPa hydrogen pressure and 150°C for 2 h. Platinum, in particular, has drawn recent attention for the hydrogenation of FF over SiO_2_ (Castelbou et al., 2017), γ-Al_2_O_3_ (Taylor et al., 2016), and TiO_2_ (Mäki-Arvela et al., 2005) oxide support. The TiO_2_-ZrO_2_ mixed oxide has a large surface area, strong surface acidity and alkalinity, high thermal stability and strong mechanical strength (Wang et al., 1983). Castelbou et al. (2017) utilized bimetallic Pt-Re supported on titanium dioxide and zirconium dioxide to catalyze the reduction of FF to FFA, with ethanol as solvent. 100% FF conversion with FFA yield of 95.7% was obtained under the reaction conditions of 130°C and 5.0 MPa hydrogen pressure (Castelbou et al., 2017). Balakrishnan et al. (2012) reported platinum, which was supported by carbon, aluminum oxide, or stannum, could catalyze the hydrogenation of 5-hydroxymethylfurfural to BHMF, and 82% yields of BHMF could be obtained with Pt_1_Sn_1_/Al_2_O_3_ at 60°C for 5 h, showing that the stannum dopant enhances its catalytic activity and selectivity for the formation of BHMF. Shi et al. (2015) prepared PtSn/SnO_2_/RGO was by first coating onto the surface of reduced graphene oxide (RGO) and SnO_2_ nanoparticles were then irradiated by microwave for a few minutes on the RGO plate. The resulting Pt_3_SnNPs efficiently catalyzed the hydrogenation of 5-hydroxymethylfurfural to BHMF, and a BHMF yield of 99% with a 99% conversion of 5-hydroxymethylfurfural was achieved at 2 MPa H_2_ under 70°C for 0.5 h, demonstrating that SnO_2_ can stabilize Pt nanoparticles and increase their catalytic activity.

The reused and regenerative properties of the catalyst played a crucial role in the choice of commercial applications. The catalyst was first reused without regeneration to determine if the activity of each reaction cycle was reduced. This indicated a slight decrease in the conversion of FF and 5-hydroxymethylfurfural per hydrogenation cycle. Similarly, it has recently been reported that other platinum-based bimetals, such as Pt-Fe/MWNT (Lesiak et al., 2014), Pt(3)Co(3)/C (Dohade and Dhepe, 2017), Pt-Sn/SiO_2_ (Liu et al., 2018), and PtCo/HCS (Wang et al., 2014) could catalyze the efficient hydrogenation of FF and 5-hydroxymethylfurfural to FFA and BHMF, respectively with activity being only slightly reduced after the repeated use. Based on this, Chatterjee et al. (2014) prepared a Pt/MCM-41 catalyst by hydrothermal method and used this catalyst to catalyze the reduction of 5-hydroxymethylfurfural to BHMF. A BHMF yield of 99% and a 100% conversion of 5-hydroxymethylfurfural was achieved at 2 MPa H_2_ under 35°C for 2 h. Recycling experiments showed that the catalyst could be recycled for at least six times, suggesting a moderate decrease in the activity possibly due to slight changes in the structure of the catalyst and Pt particle size.

#### Ru-Based Catalyst

Ruthenium-based catalysts are well-known for their excellent catalytic properties in the reduction of aromatic hydrocarbons (Cui et al., 2016), α,β-unsaturated aldehydes (Hájek et al., 2004), and furan compounds, particularly FF or 5-hydroxymethylfurfural (Nagpure et al., 2015). The monometallic Ru-based catalyst can efficiently reduce FF to furfuryl alcohol under mild conditions with temperature range and hydrogen pressure range of 20–180°C and 0.5–1.25 MPa, respectively (Table 2). The selection of the supporter becomes critical because of the particle size of ruthenium, the electron interaction of the ruthenium particles with the support, and the high reducibility of the RuO_*x*_ substance, etc., which are the key factors affecting the activity of the catalysis. Many researchers have used support, such as silica (Chen et al., 2013), carbon (Gupta and Saha, 2017), metal organic framework (MOF) (Yuan et al., 2015b), aluminum oxyhydroxide (Han et al., 2016) and magnesium oxide (Alamillo et al., 2012), etc., with different compositions and properties, to prepared ruthenium-based catalysts that were used to catalyze the reduction of FF and 5-hydroxymethylfurfural to FFA or BHMF. Among them, a carrier capable of interacting effectively with Ru particles is an ideal choice. MOFs are well-known for their strong host-guest interaction between the framework and metal particles. Yuan et al. (2015b) utilized Ru/Zr-MOFs, which prepared by deposition-reduction of Ru on the Zr-MOFs, to catalyze the hydrogenation of FF to FFA, and an FFA yield of 94.3% was obtained at a low temperature of 20°C for 4 h under low hydrogen pressure of 0.5 MPa. After the sixth recycle, the catalyst showed no decrease in the activity, possibly due to the small Ru nanoparticles size and the good reducing ability which can be attributed to the medium interaction between Zr-MOFs and Ru particles.

Many studies have displayed that ruthenium-based catalytic systems, such as Ru/Cu Nanoparticles (Zhang et al., 2019), Ru/C (Gupta and Saha, 2017), RuCo/C (Gao et al., 2016), Ru/Co_3_O_4_ [147] (Wang T. et al., 2017), Ru/Al_2_O_3_ (Alamillo et al., 2012), Ru/C+AlCl_3_ (Panagiotopoulou et al., 2015), Ru/ZrO_2_-based (Han et al., 2016), and Cm^*^Ru(HTsDPEN) (Thananatthanachon and Rauchfuss, 2010), are highly efficient for the formation of BHMF. The Ru-based catalyst for the hydrogenation of 5-hydroxymethylfurfural to BHMF under mild conditions (temperature ranged from 25 to 210°C) in the presence of different H-donors, such as H_2_ (pressures changed from 0.4 to 2.7 MPa), alcohol, or formic acid. The best catalytic activity was obtained over Ru(OH)_*x*_/ZrO_2_, affording a BHMF yield of 99% and 5-hydroxymethylfurfural conversion of 100% at 120°C for 6 h with 1.5 MPa H_2_ pressure (Han et al., 2016). The stability of heterogeneous catalysts in a continuous catalytic process is very important. After the catalyst was reused for five times, no significant loss of catalytic activity was observed, and the conversion of 5-hydroxymethylfurfural and the yield of BHMF were the same as those with the fresh Ru(OH)_*x*_/ZrO_2_ catalyst. Also, researchers added solid support, such as silica and magnesium dioxide, by different methods onto the basis of Ru/ZrO_2_-based catalysts, trying to tune the structure of the catalyst to improve its catalytic activity and stability.

Mesoporous silica is ideal support because of its large pore size and is suitable for large-sized organic substrates and biomass derivatives (Huang et al., 2011). Metal-precursor ligands are typically used to immobilize clusters in mesopores at lower metal loading due to their relatively stable nature (Mihalcik and Lin, 2008). Because the pore size of mesoporous silica (>2 nm) is larger than the particle size of metal cluster, the impregnated support cannot achieve uniform mesoporous-silica supported noble metal cluster in metal saline solution. Metal clusters readily grow into large particles with a wide distribution of size (Yen et al., 2011). It has been developed to mix mixed elements (Zr, Ti, Al, etc.) into mesoporous silica to disperse metal species. Therefore, Chen et al. reported that ruthenium clusters immobilized in nanosized mesoporous zirconium silica (MSN-Zr) were synthesized by the immersion method starting from RuCl_3_ aqueous solution. Ruthenium cluster catalysts showed significant activity for 5-hydroxymethylfurfural hydrogenation in the water at room temperature under 0.5 MPa hydrogen pressure, giving 98.1% 5-hydroxymethylfurfural conversion, and 90.4% BHMF yield (Chen et al., 2013). Alamillo et al. (2012) reported that Ru/ZrO_2_-MgO could catalyze the reduction of 5-hydroxymethylfurfural to BHMF at 130°C for 2 h, with a 5-hydroxymethylfurfural conversion of 99% and a BHMF yield of 93.1%.

#### Ir-Based Catalysts

Iridium-based catalysts can catalyze the hydrogenation of FF or 5-hydroxymethylfurfural into FFA or BHMF at a temperature ranging from 30 to 100°C, respectively, as shown in Table 2. Tamura et al. used an Ir-ReO_*x*_/SiO_2_ to catalyze the reduction of FF and 5-hydroxymethylfurfural, giving FFA or BHMF yield of 100% under the reaction conditions of 30°C for 6 h under the hydrogenation pressure of 0.8 MPa H_2_ (Tamura et al., 2013). This high catalytic performance is possibly ascribed to the cooperative effect between the iridium and the inexpensive Re(III)O_*x*_ clusters, which partially covered the iridium surface. ReO_*x*_ was believed to be high-efficiency in the reduction of polarized double bonds, promoting the adsorption of H_2_ onto the catalyst, and making it easier to dissociate the heterogeneous species of H_2_ into ionic hydrogen species (H^+^ and H^−^) (Guan et al., 2005).

Studies on the catalytic activity of Ir-based catalysts for the reduction of FF and 5-hydroxymethylfurfural by catalytic transfer hydrogenation have also been reported. Wang et al. (2015) reported that the Ir@CN catalyst, which were prepared by pyrolysis of 1,10-phenanthroline on activated carbon by pyrCl_3_ complexes, yielding small IrNPs (2–4 nm) supported on N-doped carbon, could catalyze the reduction of FF by catalytic transfer hydrogenation in the aqueous phase, affording a FFA yield of 99% at 100°C with formic acid which is an important and renewable by-product (also an H-donor) derived from the decomposition of biomass-derived carbohydrates into the levulinic acid reaction by acid catalysis (Weingarten et al., 2013).

**Scheme 3:**
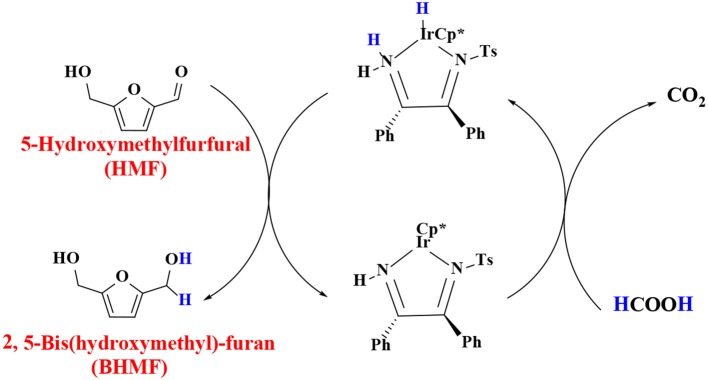
The catalytic mechanism for the hydrogenation of 5-hydroxymethylfurfural (HMF) with isopropyl alcohol using Cp^*^Ir(TsDPEN).

**Scheme 4:**
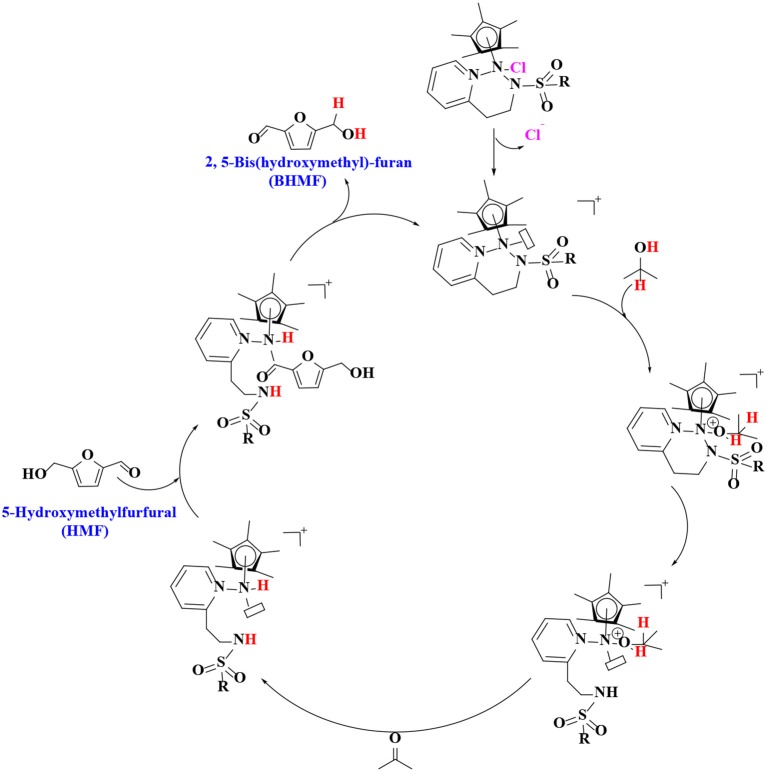
The catalytic mechanism for the hydrogenation of 5-hydroxymethylfurfural (HMF) with formic acid using Cp*Ir(pyridinesulfonamide)Cl.

In order to verify the feasibility and availability of catalytic transfer hydrogenation in other catalysts for the selective hydrogenation of 5-hydroxymethylfurfural, Thananatthanachon and Rauchfuss (2010) reported various amine–metal complexes, such as Cp^*^Ir(HTsDPEN), Cp^*^Ir(HTsDACH), and Cp^*^Ir(NHCPh_2_C_6_H_4_) (Cp^*^ = 1,2,3,4,5-pentamethylcyclopenta-1,3-diene), to catalyze the hydrogenation of 5-hydroxymethylfurfural by adopted formic acid as a hydrogen donor. Notably, up to 99% BHMF yield was achieved at 40°C. The results showed that catalytic transfer hydrogenation can be used to selectively hydrogenate 5-hydroxymethylfurfural into BHMF. Unfortunately, homogeneous catalysts, peculiarly Cp^*^Ir (HTsDACH) and Cp^*^Ir (NHCPh_2_C_6_H_4_), have poor tolerance to formic acid, a significant loss of their initial catalytic activity within a few minutes. The mechanism of the reaction demonstrates the release of carbon dioxide (Scheme 3). In order to avoid the use of formic acid, Townsend et al. (2017) used Cp^*^Ir(pyridinesulfonamide)Cl (Cp^*^ = pentamethylcyclopentadienyl) to catalyze the reduction of 5-hydroxymethylfurfural with isopropyl alcohol as hydrogen source, achieving a BHMF yield of 100% under the reaction conditions of 85°C for 0.5 h without alkali. The proposed catalytic mechanism is shown in Scheme 4. Preliminary mechanism study showed that the active catalyst for transfer hydrogenation is produced by dissociation of chloride. It is probably cooperative catalysis between the iridium center and the sulfonamide portion of the ligand.

#### Au-Based Catalysts

As a result of the low activation/dissociation potency of a hydrogen source, the reactivity mediated by Au in H-donors (e.g., H_2_ or isopropanol) is usually lower than that of the platinum group metals discussed above (Cárdenas-Lizana and Keane, 2013). The surface properties of the support, such as the surface lattice structure, surface area or acidity and alkalinity, affect the structure and electronic state of the Au species. In order to adjust the catalytic effect, it is common to add another metal oxide to the Au-based catalyst (Akita et al., 2013). For example, Zhu et al. (2016) used Au/ZrO_2_, which was synthesized by an ultrasonic auxiliary deposition method, catalyzed the hydrogenation of FF to FFA. Precipitously, Au/ZrO_2_ revealed remarkable reduction reactivity, both the conversion of FF and the yield of furfuryl alcohol reached 100% in 3 h (Zhu et al., 2016). Although Au nanoparticles are widely used as active and highly selective catalysts in oxidation reactions (Yoskamtorn et al., 2014), their use in H_2_-mediated reduction reactions is rare because H_2_ is difficult to adsorb onto the Au surface for activation (Lu et al., 2014). However, in the catalytic transfer hydrogenation process, the active H^*^ substance produced from isopropanol can promote the hydrogenation of FF at the Au surface site. In addition, it has been reported that the gold surface acted as a Lewis acid site and coupled with Lewis base to construct an effective inhibited Lewis activity on H ^*^ species by calculation and experiment (Lu et al., 2014). In the reaction system of Au/ZrO_2_, the carrier ZrO_2_ can be used as a Lewis base and formed an effective inhibited Lewis pair with the Lewis acid on the Au surface, thus enhancing the hydrogenation performance of FF. Alkaline metal oxide support can improve the selectivity of gold-based catalysts for hydrogenation of 5-hydroxymethylfurfural to BHMF (Ohyama et al., 2013).

Although the conversion of 5-hydroxymethylfurfural or FF is high, the selectivity of BHMF or FFA is very low over Pt (Balakrishnan et al., 2012), Pd (Date et al., 2017) and Cu (Srivastava et al., 2017) metal catalysts supported by Al_2_O_3_ in the reduction of 5-hydroxymethylfurfural. Therefore, Ohyama et al. (2013) utilized Au supported by trialumina to catalyze the selective reduction of 5-hydroxymethylfurfural to BHMF. Both the conversion of 5-hydroxymethylfurfural and the yield of BHMF were >96% under the reaction conditions of 120°C and 6.5 MPa H_2_ for 2 h. The high catalytic activity of Au/Al_2_O_3_ for catalytic reduction of 5-hydroxymethylfurfural to BHMF is attributed to the high hydrogenation activity of small Au clusters and the difficulty in converting BHMF to furan-ring opening products for its unique acid-base properties. However, the reaction conditions for the highly efficient and highly selective reduction of 5-hydroxymethylfurfural to BHMF by Au/Al_2_O_3_ are harsh. For example, both the reaction temperature and hydrogen pressure are high (120°C, 6.5 MPa). In order to make Au/Al_2_O_3_ hold excellent catalytic performance under mild reaction conditions, Ohyama et al. doped iron oxides with the support Al_2_O_3_, and the resulting catalyst (Au/FeO_*x*_/Al_2_O_3_) is highly selective for the reduction of 5-hydroxymethylfurfural to BHMF, giving 5-hydroxymethylfurfural conversion and BHMF yield both in 96% at 80°C and 3 MPa H_2_ for 2 h (Ohyama et al., 2016).

**Scheme 5:**
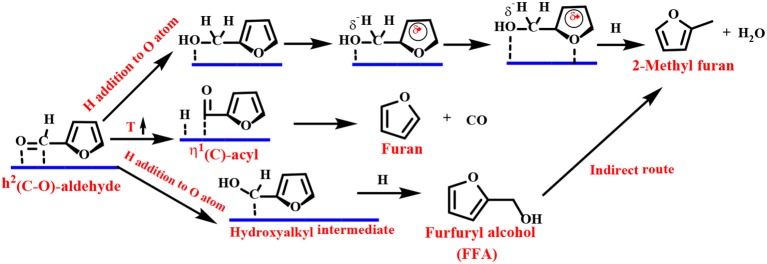
Schematic illustration of FF hydrogenation to furfuryl alcohol (FFA) over group VIII metal-based catalysts.

The Group VIII metal (Pd, Pt, Ir, Ru, or Au) does not have repulsion between the furan ring and the metal surface and favors a flat η^2^ (C–O) bonding mode. The preferred basic route for FFA is carried out in two steps (Scheme 5): (1) the hydrogenation reaction flat adsorbs the carbonyl group of η^2^ (C–O)-FF to form a hydroxyalkyl intermediate, followed by (2) reduction of the carbon with a hydroxyl group to form FFA.

Generally, various noble metals (e.g., Pd, Pt, Ru, Ir, and Au) and non-noble metals, including different chromium and non-chromium metals [such as Cu- and ferrous metals (Fe, Ni, or Co)-based], have been reported for the selective hydrogenation of FF and 5-hydroxymethylfurfural in the gas phase and the liquid phase. Chromium catalysts show good catalytic activity, but they are not widely available due to their high toxicity. The non-chromium metals, such as Cu-based catalysts exhibit a remarkable selectivity for the hydrogenation of a carbonyl group, and the C–C double bond in the furan ring does not participate in the reaction. A second metal or promoter is sometimes added to improve activity or selectivity by increasing the surface area or acting as a Lewis acid site to polarize the C–O bond. At the same time, the iron/cobalt/nickel-based catalysis also achieved better catalytic effects. However, the main drawback is that most of these catalysts cannot be reused. Therefore, other noble metals, such as Pd, Pt, Ru, Ir, and Au, were added to increase their stability, where ethanol, water, ethanol, isopropyl alcohol, *n*-butyl alcohol, benzyl alcohol, heptane, tetrahydrofuran, or octane was used as the solvent. The mechanism of precious and non-precious metal particles catalyzed the reduction of FF and 5-hydroxymethylfurfural to FFA and BHMF is also described.

### Solid Acid-Base Catalysts

Solid acid-base catalysts, such as metal hydroxides and solid Lewis acid/base materials, are widely used in the reduction of FF and 5-hydroxymethylfurfural by catalytic transfer hydrogenation. Solid catalysts containing Lewis acids are often used for intermolecular and intramolecular hydrogen transfer, so many researchers use them to catalyze the upgrading reaction of biomass to produce valuable compounds (Li et al., 2014). The use of liquid hydrogen sources (e.g., alcohol and formic acid) instead of high-pressure H_2_ addresses the safety hazards posed by hydrogen (He et al., 2019b). In relation to this, it has recently been reported that Lewis acid zeolites, such as Hf- and Zr-, are used for the hydrogenation of biomass-derived carboxides, such as FF and 5-hydroxymethylfurfural, into corresponding alcohols through the reduction of Meerwein-Ponndorf-Verley (MPV), as shown in Table 3. For example, Hao et al. (2016) reported that ZrO(OH)_2_, which contained acidic and basic sites, catalyzed the hydrogenation of 5-hydroxymethylfurfural to BHMF, giving 83.7% yield of BHMF by the catalytic transfer hydrogenation reaction. This interesting work prompted more researchers to explore the complex containing zirconyl acid-base coupling site for catalytic transfer hydrogenation reaction, which could not only overcome the drawbacks encountered in the catalytic process (e.g., catalyst deactivation and harsh reaction conditions) but also improve the catalytic performance of the Zr-based materials. It is well-known that the magnetic catalysts can be easily separated from the reaction mixture by an external magnet for its distinct properties. Therefore, Hu et al. (2018) added magnetic components, such as iron, to zirconium hydroxide to prepare magnetic zirconium hydroxides MZH (Zr/Fe = 2), which was used to catalyze the reduction of 5-hydroxymethylfurfural to BHMF at 150°C for 5 h using 2-butanol as hydrogen source, and the obtained conversion of 5-hydroxymethylfurfural and the yield of BHMF were up to 98.4 and 89.6%, respectively. The catalyst could be recycled for continuous 5 times with no significant decrease in catalytic activity (Hu et al., 2018). Inorganic-organometallic phosphonates are well-known for their unique properties, such as excellent hydrolytic stability for silicas and polymers (de los Reyes et al., 2013). They are used in a wide range of applications for the adsorption and separation of toxic and contaminants, energy storage, and drug delivery (Ma and Yuan, 2011), and being used as a catalyst (Bhanja and Bhaumik, 2016) in the reduction of biomass-based aldehydes, especially FF and 5-hydroxymethylfurfural.

**Table 3:** Reduction of FF and 5-hydroxymethylfurfural catalyzed by various acid-base catalysts.

**Substrate**	**Catalyst**	**Reaction condition**			**H-donor**	**Main product**	**Catalytic activity**			**Reusability**				**References**
		**Solvent**	**Temp. (°C)**	**Time (h)**			**Conv. (%)**	**Yield (%)**	**Sel. (%)**	**Cycles**	**Yield (%)**	**Sel. (%)**	**Conv. (%)**	
HMF	ZrO(OH)_2_	Ethanol	150	2.5	Ethanol	BHMF	94.1	83.7	89	Six	47	63.2	74.9	Hao et al., 2016
HMF	MZH(Zr/Fe = 2)	2-butanol	150	5	2-butanol	BHMF	98.4	89.6	91	Five	87	90	97	Hu et al., 2018
FF	ZrPN	Isopropanol	140	2	Isopropanol	FFA	98	98	100	NM	NM	NM	NM	Li et al., 2016a
HMF	ZrPN	Isopropanol	140	2	Isopropanol	BHMF	99	98	99	NM	NM	NM	NM	Li et al., 2016a
HMF	Zr-BPPN	Isopropanol	120	2	Isopropanol	BHMF	99	93	94	NM	NM	NM	NM	Li et al., 2017a
FF	Zr-FDCA	Isopropanol	140	8	Isopropanol	FFA	98	96	98	Six	97	94	–	Li et al., 2017b
HMF	Zr-FDCA	Isopropanol	140	8	Isopropanol	BHMF	97	87	90	NM	NM	NM	NM	Li et al., 2017b
FF	PhP-Hf (1:1.5)	Isopropanol	120	2	Isopropanol	FFA	100	99.2	99.2	Five	92.3	98.8	99.2	Li et al., 2019
HMF	Hf-FDCA	Isopropanol	100	5	Isopropanol	BHMF	–	95	–	NM	NM	NM	NM	Li et al., 2018a
FF	Hf-MOF-808	Isopropanol	100	2	Isopropanol	FFA	–	97	–	Five	80	–	–	Rojas-Buzo et al., 2018
HMF	Hf-MOF-808	Isopropanol	100	1.5	Isopropanol	BHMF	–	92	–	NM	NM	NM	NM	Rojas-Buzo et al., 2018
HMF	ZrBa(3)-SBA	Isopropanol	150	2.5	Isopropanol	BHMF	98.3	91	92.2	Five	81.8	100	81.6	Wei et al., 2018
FF	MgO	Isopropanol	170	5	Isopropanol	FFA	>99.9	74	74	Five	74	74	>99.9	Biradar et al., 2016
HMF	MgO	Methanol	160	3	Methanol	BHMF	100	100	100	NM	NM	NM	NM	Pasini et al., 2014
FF	γ-Fe_2_O_3_@HAP	Isopropanol	180	3	Isopropanol	FFA	90	65	72	Six	63	70	90	Wang and Zhang, 2016
FF	γ-Fe_2_O_3_@HAP	Isopropanol	180	10	Isopropanol	FFA	96.2	91.7	95.3	NM	NM	NM	NM	Wang and Zhang, 2016
FF	Al_7_Zr_3_@Fe_3_O_4_ (1/1)	Isopropanol	180	4	Isopropanol	FFA	99.1	90.5	91	Five	83.8	87	95.9	He et al., 2018a
HMF	Al_7_Zr_3_@Fe_3_O_4_	Isopropanol	180	4	Isopropanol	BHMF	82.7	71	86	NM	NM	NM	NM	He et al., 2018a

*PN, Organotriphosphate; FDCA, 2,5-Furandicarboxylic acid; BPPN, Benzylphosphonate nanohybrids*.

It is desirable that the pore size of the catalyst, which can be controlled by mesopores and macropores, ensures that the substrate readily enters the active site during the catalytic process. (Li et al., 2016a) reported that ZrPN (organotriphosphate-zirconium complex), a heterogeneous nitrogen-containing alkyltriphosphonate-metal hybrid (MPN), which can be used to enhance acid and base functionalization, and prepared by a simple method with no additional addition of a template. Proper adjustment of the alkali/acid site ratio in ZrPN can enhance the catalytic performance of the catalyst in the hydrogenation of FF and 5-hydroxymethylfurfural to FFA or BHMF, respectively. The yield of FFA and BHMF was significantly improved when the base/acid site is 1:0.7, FF conversion and FFA yield both in 98% were obtained at 140°C for 2 h by using isopropanol as H-donor (Li et al., 2016a). Under the same reaction conditions, the conversion of 5-hydroxymethylfurfural and the yield of BHMF reached 99 and 98%, respectively. Then, Li et al. (2017a) used the same method for synthesizing Zr-benzylphosphonate nanohybrids (BPPN), which can be prepared from *o*-, *p*-, or *m*-xylylenediphosphonates (*o*-, *p*-, or *m*-PhP) and zirconium salt via a simple and template-free assembly method. The conversion of 5-hydroxymethylfurfural and the yield of BHMF reached 99 and 93% over Zr-BPPN at 120°C for 2 h by using isopropanol as H-donor, respectively (Li et al., 2017a).

Metal-organic frameworks (MOFs), which were usually composed of metal substances and organic ligands, are known for the ability to produce porous and crystalline polymers, showing great potential in various fields, such as separation of materials, gas adsorption, sensing, storing of energy, and delivering of drug due to their unique properties (Zhou and Kitagawa, 2014). More and more researchers are keen to develop functional and structural materials for natural composites in crucial areas, such as material chemistry (Wegst et al., 2015). It is reported that substantial porous carbonaceous materials can be prepared by calcining and modifying lignocellulose with different functional groups, and showed very strong application prospects in many important fields, such as catalysis and drug delivery (Kai et al., 2016). The relatively strong stability of functional and structural materials can be obtained from natural organic molecular materials, such as phytic acid, polyphenols, and porphyrins, by self-assembly with a metal ion, such as Zr^4+^ (Song et al., 2015). 2,5-furandicarboxylic acid (FDCA), whose structure is similar to terephthalic acid, contains two –COOH groups at the 2,5-position of the ring. FDCA can also be used as an excellent organic ligand for the preparation of MOFs because it is beneficial to the production of polymers (Han et al., 2017). Inspired by these findings, Li et al. (2017a) prepared a mesoporous acid-based bifunctional nanocomposite Zr-FDCA by hydrothermal treatment of FDCA with zirconium ion, and the obtained Zr-FDCA was highly efficient for the reduction of biomass-based aldehydes, especially FF and 5-hydroxymethylfurfural, giving FF and 5-hydroxymethylfurfural conversion of 98 and 97% with FFA and BHMF yield of 96 and 87% at 140°C for 8 h by utilizing isopropanol as hydrogen source, respectively (Li et al., 2017a). Considering that the reusability of a solid catalyst is one of the most important features to evaluate its potential application in industrial production (Garcia-Olmo et al., 2016), Zr-FDCA was reused for six consecutive cycles and no significant loss in reactivity was observed, with 98% FF conversion and 96% FFA yield in the sixth cycle.

Besides FDCA, Li et al. (2019) also used *para*-xylylenediphosphonates as ligands to prepare Hf-based MOFs (PhP-Hf) by self-assembly with hafnium ion, and used for the hydrogenation of FF and 5-hydroxymethylfurfural. PhP-Hf (1:1.5) could catalyze the hydrogenation of FF to FFA, affording FF conversion of 100% with FFA yield of 99.2% at 120°C for 2 h using isopropanol as the hydrogen source. After the catalyst being reused for 5 times, only a minor decrease in catalytic activity was observed, indicating that the stability of the catalyst was high (Li et al., 2019). Similarly, Hf-FDCA prepared with the same method could also catalyze the reduction of 5-hydroxymethylfurfural to BHMF, achieving a high BHMF yield of 95% at 100°C for 5 h by using isopropanol as hydrogen source (Li et al., 2018a).

**Scheme 6:**
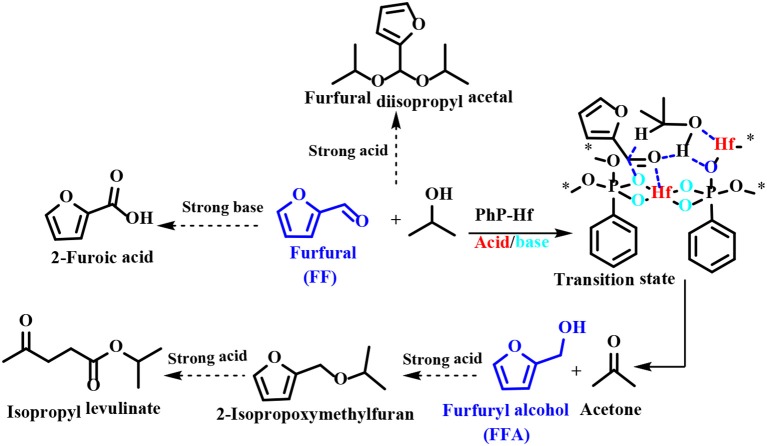
A possible mechanism for transfer hydrogenation of FF to FFA catalyzed by PhP-Hf (1:1.5).

Regarding the reaction mechanism of PhP-Hf, the acid site (Hf^4+^) and the base site (O^2−^) of PhP-Hf (1:1.5) proved to synergistically catalyze the reduction of FF to FFA by MPV reaction. It was proposed that the critical transition state shown in Scheme 6 is *in situ* formed among FF, isopropanol, and acid-base coupling materials (Hf^4+^-O^2−^). Typically, a pair of acid-base sites (Hf^4+^-O^2−^) facilitates the adsorption of isopropanol onto PhP-Hf (1:1.5) and is capable of producing isopropanol and hydride by dissociation. The aldehyde groups in the FF may be activated by acidic Hf^4+^ species, thus resulting in the formation of a transition state with six linkages to achieve a hydrogen transfer pathway to produce FFA and acetone (Scheme 6). To be noted, some by-products, such as FF diisopropyl acetal, 2-isopropoxymethylfuran, isopropyl levulinate, and 2-decanoic acid may be formed in the presence of relatively strong acidic and basic sites.

Rojas-Buzo et al. (2018) used Hf-MOF-808 catalyst, which was prepared by self-assembly of 1,3,5-benzenetricarboxylic acid and HfCl_4_, to catalyze the reduction of FF and 5-hydroxymethylfurfural utilizing isopropanol as H-donor, giving FFA and BHMF both in yield of 97% at 100°C for 2 and 1.5 h, respectively. No significant loss in reactivity was found after the catalyst reused for 5 times, indicating that this catalyst was relatively stable. In the hydrogenation of FF and 5-hydroxymethylfurfural, the Zr-MOF or Hf-MOF catalyst not only exhibited excellent selectivity toward the formation of FFA and BHMF, but also showed relatively high catalytic stability during recycles. Zr-Beta or Sn-Beta zeolite catalyst also exhibits excellent catalytic performance during the hydrogenation of 5-hydroxymethylfurfural to BHMF via catalytic transfer hydrogenation using isopropanol as a hydrogen source. The strong Lewis acid sites on these catalysts can effectively catalyze the reduction of 5-hydroxymethylfurfural to BHMF, and also catalyzed the etherification of BHMF (Luo et al., 2014). The Lewis or Brønsted acid sites can be effectively adjusted by adding active metal oxide to the Zr-based catalyst. Wei et al. (2018) reported ZrBa(3)-SBA, prepared by loaded active metal oxides using the successive incipient-wetness impregnation method, showed high catalytic activity in the chemoselective reduction of 5-hydroxymethylfurfural to BHMF, with the 5-hydroxymethylfurfural conversion of 98.3% and BHMF yield of 91%. Exceptionally, ZrBa-SBA can be reused continuously for five times with no significant loss of catalytic performance, indicating that it was a stable catalyst for the tested reaction. ZrBa(3)-SBA exhibits high catalytic activity, which is attributable to the significant reduction in the total acid sites of ZrBa-SBA by the introduction of BaO, completely suppressing the etherification of 5-hydroxymethylfurfural or BHMF. However, the remaining acid sites of ZrBa-SBA (Lewis acid) are still sufficient for selective reduction of 5-hydroxymethylfurfural (Wei et al., 2018).

Typically, the preparation of Lewis acidic zeolites, such as Zr^4+^ catalysts is cumbersome. On the other hand, the presence of a strong Lewis acid causes some side reactions, such as etherification of FFA or BHMF with an alcohol, and ring opening of the furan ring. Therefore, it is still necessary to develop new catalytic pathways for the effective conversion of FF and 5-hydroxymethylfurfural to FFA and BHMF, respectively. It has been reported that base catalysts have great potential for catalyzing the carbonyl compounds to corresponding alcohols via catalytic transfer hydrogenation (Wang and Zhang, 2016). Biradar et al. (2016) showed that magnesium oxide could catalyze the hydrogenation of FF to FFA. Both the conversion of furfural and the yield of FFA were 99.2% at 170°C for 5 h utilizing isopropanol as the hydrogen source. MgO could be reused continuously for 5 cycles without significant loss in catalytic activity. Pasini et al. (2014) also used magnesium oxide as a catalyst for the hydrogenation of 5-hydroxymethylfurfural using methanol as a clean and efficient hydrogen source, affording 5-hydroxymethylfurfural conversion and BHMF yield both in 100% at 160°C for 3 h. Inexpensive mixed metal oxides are broadly used in catalytic fields due to their adjustable physicochemical properties, such as surface area, pore size distribution, and acid/base characteristics, which are closely related to catalytic activity. Solid materials with magnetic properties are the most interesting catalyst supporters due to their unique properties. For example, magnetic catalysts can be separated from the reaction mixture by external magnets due to their unique properties, which avoid weight loss of the catalyst during repeated use and make the operation of this catalytic system time-saving and energy efficient (Vu et al., 2014). Wang and Zhang (2016) supported γ-Fe_2_O_3_ onto hydroxyapatite to prepare γ-Fe_2_O_3_@HAP catalyst, which was used as a magnetic base catalyst to catalyze the reduction of FF into FFA. The conversion of FF and the yield of FFA were 90 and 65% at 180°C for 3 h by utilizing isopropanol as the hydrogen source, respectively. After recycling for 6 times under the optimized reaction conditions, the catalytic activity was not reduced. When the reaction time was extended to 10 h, the conversion of FF and the yield of FFA were up to 96.2 and 91.7%, respectively (Wang and Zhang, 2016). He et al. (2018a) also reported another magnetic catalyst Al_7_Zr_3_@Fe_3_O_4_, an acid/base bi-functional magnetic oxide catalyst, which can be prepared by a facile co-precipitation method. Al_7_Zr_3_@Fe_3_O_4_ could catalyze the hydrogenation of FF to FFA by using isopropanol as a hydrogen source, giving FF conversion of 99.1% and FFA yield of 90.5% at 180°C for 4 h. Al_7_Zr_3_@Fe_3_O_4_ (1/1) acted as a heterogeneous catalyst and could be recycled for 5 times with no remarkable loss of catalytic performance. Moreover, it was found that the weight of the catalyst was not lost after a straightforward recovery by using an external magnet. Remarkably, the catalyst also showed high-efficiency for reduction of other biomass-derived furanic aldehydes, such as 5-hydroxymethylfurfural. The conversion of 5-hydroxymethylfurfural and the yield of BHMF were 82.7 and 71% under the optimized reaction conditions, respectively (He et al., 2018a).

In general, solid acid/base catalysts, such as ZrO(OH)_2_, Zr- or Hf-MOF, Zr- or Sn-Beta zeolite, MgO, γ-Fe_2_O_3_@HAP, and Al_7_Zr_3_@Fe_3_O_4_, were able to efficiently catalyze the hydrogenation of 5-hydroxymethylfurfural and FF to corresponding alcohols via MPV reduction. Zr- and Hf-MOF catalysts not only exhibited excellent selectivity for the synthesis of FFA or BHMF, but also showed relatively high catalytic stability during recycles. Zr- or Sn-Beta zeolite catalyst also exhibits excellent catalytic performance during the hydrogenation of 5-hydroxymethylfurfural to BHMF via catalytic transfer hydrogenation using isopropanol as the hydrogen source. Inexpensive mixed metal oxides, such as MgO and γ-Fe_2_O_3_@HAP, also achieved the good catalytic activity. Further, the mechanism mediated by the solid acid/base catalyst through the reduction of MPV was discussed.

### Alkali Salt Catalysts

As discussed above, there have been many reports on different types of catalysts including noble metals (e.g., Pd, Pt, Ir, Ru, and Au) (Lesiak et al., 2014; Date et al., 2018; Shirvani et al., 2018), non-noble metals (e.g., Cu, Ni, Fe, Mg, and Co) (Villaverde et al., 2013; Yan et al., 2013b; Khromova et al., 2016; Osatiashtiani et al., 2017), and solid acid-base bifunctional catalysts (e.g., the Zr-and Hf-based) for the hydrogenation of FF or 5-hydroxymethylfurfural. However, they are either expensive or complicated to prepare. It is worth mentioning that H_2_ is the most common source of hydrogen, but usually requires a higher pressure (e.g., 8 MPa). In particular, when it comes to gas phase hydrogenation, a series of pressure-resistant instruments are required (Chen et al., 2002). For the purpose of safety and low cost, liquid phase transfer hydrogenation has received much attention by using formic acid or an alcohol, such as isopropanol as the H-donor (Addis et al., 2011; Li et al., 2016a). However, using formic acid as the hydrogen donor, there is some corrosion to the experimental equipment, and the alcohol solvent can be further reacted with FFA by etherification to reduce its selectivity under the harsh conditions employed (Zhang et al., 2016). Therefore, it is increasingly important to find an inexpensive and safe hydrogen donor and more desirable catalysts for the hydrogenation of FF and 5-hydroxymethylfurfural. Hydrosilylation is widely used in the catalytic reaction of carbonyl compounds to the corresponding alcohols (Oestreich and Hermeke, 2015). This catalytic process is very attractive using hydrosilanes as hydrogen source, which are very stable in water and air, and can be activated to produce silyl ethers that can be converted to alcohol and silanol via hydrolysis with an acid/base catalyst or activator under relatively mild conditions (Revunova and Nikonov, 2015; Muthukumar et al., 2016). Long et al. (2018) reported that potassium carbonate, an abundant, readily available, and cost-effective salt, could catalyze the hydrogenation of 5-hydroxymethylfurfural into BHMF at room temperature using Ph_2_SiH_2_ as H-donor in the presence of bio-based solvent 2-methyltetrahydrofuran, giving a good BHMF yield of 94% after 2 h (Table 4, entry 1). However, using Ph_2_SiH_2_ as hydrogen source not only tends to produce high molecular weight by-products, such as silanols that are detrimental to product separation, but also its reserve is not too much due to the relatively expensive price (Zhao et al., 2014). It is impending to find more ideal H-donor for the hydrogenation of biofuranic aldehyde. Polymethylhydrosiloxane (PMHS) is an attractive H-donor among commercially available hydrosilanes, which can be attributed to its unique character, which is inexpensive, commercially available, non-toxic, biodegradable, and stable in air and moisture (Volkov et al., 2015). In addition, PMHS is soluble in most organic solvents due to its low viscosity (Kumar et al., 2017). Based on these findings, Long et al. (2019) depicted a benign method to effectively catalyze the reduction of FF to FFA over commercially available Cs_2_CO_3_ utilizing non-toxic and cheap PMHS as a hydrogen source, giving an excellent FFA yield of 99% (Table 4, entry 2).

**Table 4:** Reduction of FF and 5-hydroxymethylfurfural catalyzed by various alkali salt catalysts.

**Substrate**	**Catalyst**	**Reaction condition**			**H-donor**	**Main product**	**Catalytic activity**			**Reusability**				**References**
		**Solvent**	**Temp. (°C)**	**Time (h)**			**Conv. (%)**	**Yield (%)**	**Sele. (%)**	**Cycles**	**Yield (%)**	**Sele. (%)**	**Conv. (%)**	
HMF	K_2_CO_3_	2-methyltetrahydrofuran	25	2	Ph_2_SiH_2_	BHMF	100	94	94	NM	NM	NM	NM	Long et al., 2018
FF	Cs_2_CO_3_	N,N-dimethylformamide	80	6	PMHS	FFA	99	99	100	NM	NM	NM	NM	Long et al., 2019
FF	KF	N,N-dimethylformamide	25	0.5	PMHS	FFA	99	99	100	Three	87	97	90	Wu et al., 2018
HMF	KF	Dimethyl sulfoxide	25	0.5	PMHS	BHMF	>99	>98	99	Three	97	99	98	Zhao et al., 2018

**Scheme 7:**
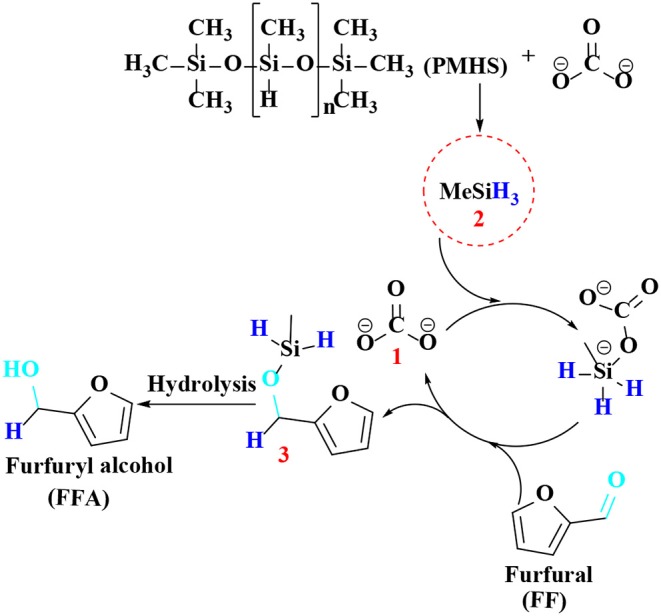
Proposed reaction pathway for the conversion of FF to furfuryl alcohol (FFA).

Regarding the reaction mechanism of Cs_2_CO_3_, Lewis base (carbonate **1**) proved to efficiently catalyze the reduction of FF to furfuryl alcohol by hydrosilylation reaction (Scheme 7). After reacting with Lewis base (carbonate **1**), PMHS is cleaved into small silane (H_3_SiMe **2**), which forms pentavalent silicate with carbonate, and then reacts with carbonyl in FF to produce a hexavalent intermediate, which can activate the aldehyde group by hydride to obtain silyl ether **3**, which is then hydrolyzed to gain FFA.

Although the above two alkali metal carbonates have excellent catalytic effects, the activity of the catalyst is greatly reduced after repeated use, which could be attributed to the interaction of carbonate with hydrosilane, facilitating the formation of silicon formate. The reusability of a solid catalyst is one of the most important features to evaluate its potential application in industrial production. Therefore, Zhao et al. (2018) utilized potassium fluoride that is a cheap alkali salt to catalyze the hydrogenation of FF and 5-hydroxymethylfurfural to FFA or BHMF, giving FFA or BHMF in a high yield of up to 97 and 98%, respectively (Wu et al., 2018; Zhao et al., 2018; Table 4, entries 3 and 4). Remarkably, this catalyst could be recycled for several consecutive cycles with no distinct loss of activity, demonstrating that the stability of potassium fluoride is high during the catalytic procedure.

## Conclusions and Perspectives

This review illustrates the performance of heterogeneous catalysts, especially the metallic particles (supported on Cu, Mg, Fe, Ni, Co, Pd, Pt, Ru, Ir, and Au metals), solid acid/base (e.g., Zr-and Hf-based), and alkali metal salts (e.g., K_2_CO_3_, Cs_2_CO_3_, and KF) in the hydrogenation of biofuranic aldehydes to alcohols. Inferred from recent reaction relationships and reaction pathways, the conversion of FF and 5-hydroxymethylfurfural to useful chemicals and biofuels by different catalysts mainly involves several processes related to hydriding, destructive hydrogenation, decarburization, oxidization, and ring opening reactions. Therefore, the catalyst design is the principal element to acquire high-efficiency conversion of FF and 5-hydroxymethylfurfural to the desired products. Based on this direction, with hydrogen as the hydrogen source, the Cu-based catalysts exhibit a stronger avidity for the hydrogenation of FF and 5-hydroxymethylfurfural to FFA or BHMF, respectively, while the hydrogenation of the C = C-bonded furan ring on the Cu-based catalysts is not affected because of the exclusion between the furan nucleus and the Cu surface. Similarly, the Fe catalyst showed an excellent effect of selective hydrogenation of FF and 5-hydroxymethylfurfural to yield FFA or BHMF. Though the Ni catalysts exhibit high performance in the hydrogenation of biofuranic compounds, it is accompanied by side reactions, leading to the hydrogenation of the double bond of the furan ring. The Pd catalysts promote hydriding of C = O and C = C bonds and destructive hydrogenation of C-O bonds. In addition, under elevated conditions, the Pd catalyst also undergoes decarbonylation of FF or 5-hydroxymethylfurfural and furan ring opening due to the friable interactions between the carboxide carbon and palladium surfaces. Pt catalyst is widely used in the hydrogenation reactions due to its unique electrical and chemical properties. Obviously, the Au catalysts also exhibit relatively high catalytic performance in the furan ring opening reaction. In addition, Ru catalysts have been shown to have high catalytic activation for hydriding of C = O bonds and then destructive hydrogenation, gaining effective and useful production with fewer O atoms.

Metal hydroxides and solid Lewis acid/base materials are widely used in the hydrogenation of FF and 5-hydroxymethylfurfural by catalytic transfer hydrogenation with efficient catalytic performance. The utilization of alkali metal salts can not only solve the complicated preparation process of the catalyst, but also avoid the use of high-pressure hydrogen, alcohol, and formic acid by using hydrogen silane as a hydrogen source. The property of the catalyst' supporter (acidity, alkalinity and neutral) also plays a crucial character in these catalytic conversion reactions. Since the acidic carrier has a great influence on the hydrogenolysis and ring opening of the furan ring, which contributes to the cleavage of the C-O bond. However, the basic carrier results in either the oxidation or over hydrogenation of the furan-ring. Furthermore, the protic solvent promotes a higher solvability of the hydrogen than the aprotic solvent, and thus enhances the catalytic activation by increasing the interaction of hydrogen with the catalyst surface. In addition, the morphology and the particle size of the catalyst also have a significant influence on the catalytic capability. Furthermore, it is known that the incorporation of a second metal plays an essential role in the geometry and electron concentration of the active site of the conditioning catalyst, which results in the bimetallic catalyst to exhibit enhanced catalytic activation and stabilization. Although precious metal catalysts exhibit enhanced catalytic performance for furan conversion, several non-precious metals (Fe, Ni, Co, and Cu) supported heterogeneous catalysts have also been investigated. Hence, in order to satisfy global demand, the rich and cheaper metal catalytic system on the earth is gaining huge scientific and industrial concerns. Moreover, studies have shown that different other reaction parameters, such as reaction temperature, exhaust gas pressure (hydrogen), solvent properties (polar or non-polar), reducing agents (H_2_, ethanol, silane) also have a great influence on the catalytic reaction. This review not only discusses the mechanism by heterogeneous catalysts for the conversion of xylose and glucose to FF or 5-hydroxymethylfurfural, but also depicts representative examples on the conversion of FF or 5-hydroxymethylfurfural to FFA or BHMF in the presence of different catalysts, respectively, providing a theoretical support for the exploration of more environmentally friendly approaches to the hydrogenation of biomass-derived carbonyl compounds.

Although significant progress has been achieved for heterogeneous catalytic upgrading of biomass-derived furanic compounds, some advances are still needed in plenty of cases for the exploration of mercantile applications:

- Design of novel multi-functional catalysts to promote interaction with various substrates, so as to promote multi-step reactions in one-pot reactions, avoiding isolation and purification of intermediates.
- Control the acidity/alkalinity of the catalyst to improve the selectivity of the desirable product.
- In addition to precious metals, the establishment of non-precious metal-based catalysts is highly desirable for upgrading biomass-derived compounds.
- To comprehend mechanism insights and the catalyst structure-activity relationship, so as to guide the development of more efficient catalysts.
- Though it is safe and inexpensive to use hydrosilane as a hydrogen source, the products are not easy to separate. It is necessary to find a more favorable hydrogen source, greener solvent or improved catalytic system/process, which is better for separation of the products with good activity.
- The direct use of lignocelluloses, a cheaper and more readily available natural source, for biomass-derived alcohols would be of particular importance, thus decreasing the release of petroleum-derived pollutants.
- To meet the multiple conversion steps for biomass upgrading, it would be highly desirable to develop more economic and facile approaches for the preparation of multifunctional catalysts that can promote cascade or complex reaction processes.

Overall, this review comments recent examples on the catalytic hydrogenation of biofuranic aldehydes to alcohols over different heterogeneous catalysts. We believe that a comprehensive study on a wide range of catalytic biomass conversion processes may help to develop new and even more efficient ways for the upgrade of biomass-derived compounds to useful chemicals and biofuels.

## Author Contributions

JL and HL: taking the lead in coordinating the review study and drafting the manuscript. JL, YX, and WZ: literature searching. SY: supervision. HL: conceptualization. SY and HL: funding acquisition and project administration. JL: writing-original draft. HL and SY: writing-review and editing.

### Conflict of Interest Statement

The authors declare that the research was conducted in the absence of any commercial or financial relationships that could be construed as a potential conflict of interest.
